# Supplementary material for: Structure of the far-red light utilizing photosystem I of Acaryochloris marina
Source: Nat Commun. 2021 Apr 20;12:2333. doi: 10.1038/s41467-021-22502-8 (PMC8058080; doi:10.1038/s41467-021-22502-8)
Supplement: Supplementary file 1 — Supplementary Information [file 41467_2021_22502_MOESM1_ESM.pdf]

Supplementary Information

Structure of the far-red light utilizing photosystem I of *Acaryochloris marina*

Tasuku Hamaguchi, Keisuke Kawakami, Kyoko Shinzawa-Itoh, Natsuko Inoue-Kashino, Shigeru Itoh, Kentaro Ifuku, Eiki Yamashita, Kou Maeda, Koji Yonekura and Yasuhiro Kashino

Correspondence to: kawakami.k@spring8.or.jp, yone@spring8.or.jp, and kashino@sci.u-hyogo.ac.jp

This file includes:

Supplementary Figures 1–22
Supplementary Tables 1–7

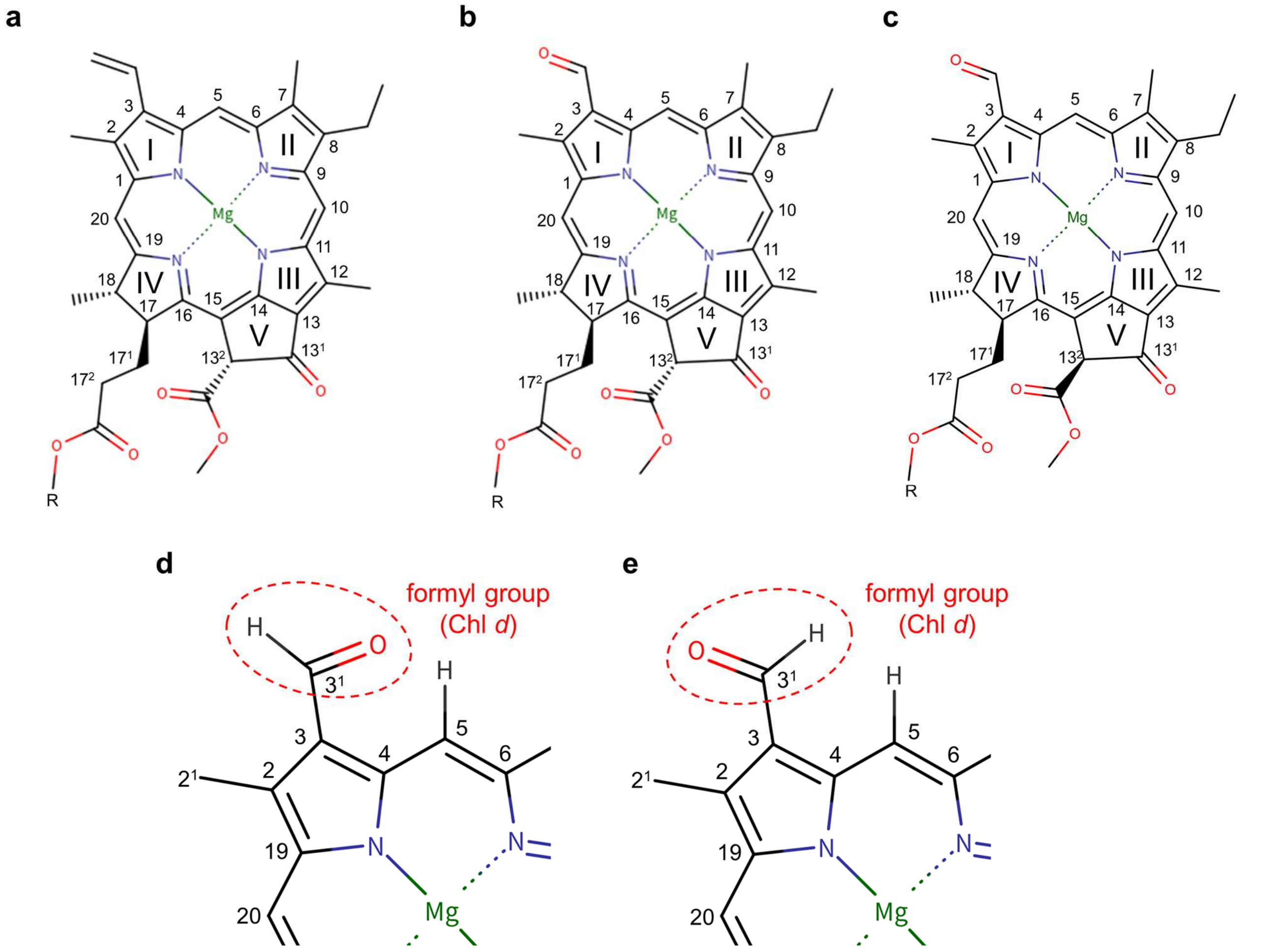

Supplementary Fig. 1: Molecular structures and carbon numbering of Chl *a* and Chl *d* according to the IUPAC numbering system (R = phytol chain).

Structure of Chl *a* (a), Chl *d* (b), and Chl *d*’ (c). Possible orientations of the formyl group in Chl *d*; (d) the carbonyl O atom is oriented toward the C5 H atom; (e) the formyl group flipped along the C3−C3^1 axis compared with (d) according to Saito *et al.*[52].

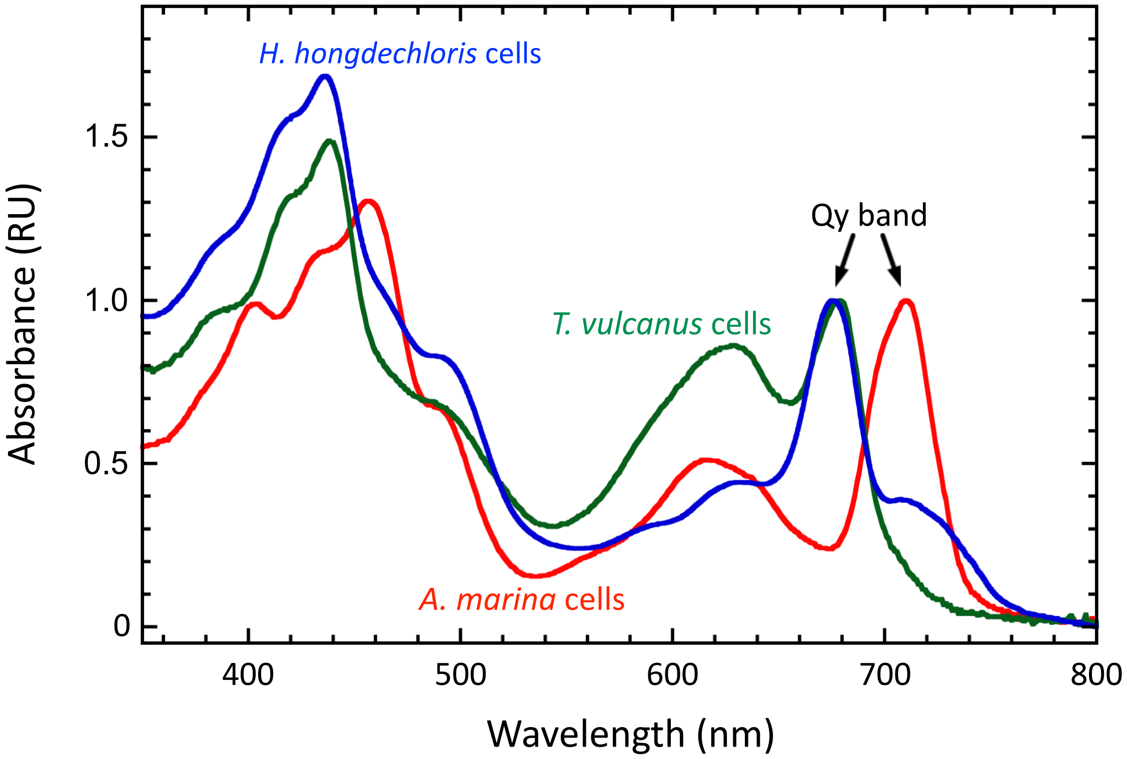

Supplementary Fig. 2: Comparison of cellular absorption spectra of three cyanobacteria. Absorption spectra of *A. marina* (red line) and *Thermosynechococcus vulcanus* (green line) cells equivalent to 8.1 µg Chl *d*/mL and 5.0 µg Chl *a*/mL, respectively, were measured with an integrating sphere. Absorption spectrum of Chl *f*-carrying *Halomicronema hongdechloris* (blue line) cells grown in far red light, which is a courtesy of Prof. Tatsuya Tomo, is also presented. Spectra were normalized by the peak height of the Qy band with offset at 800 nm.

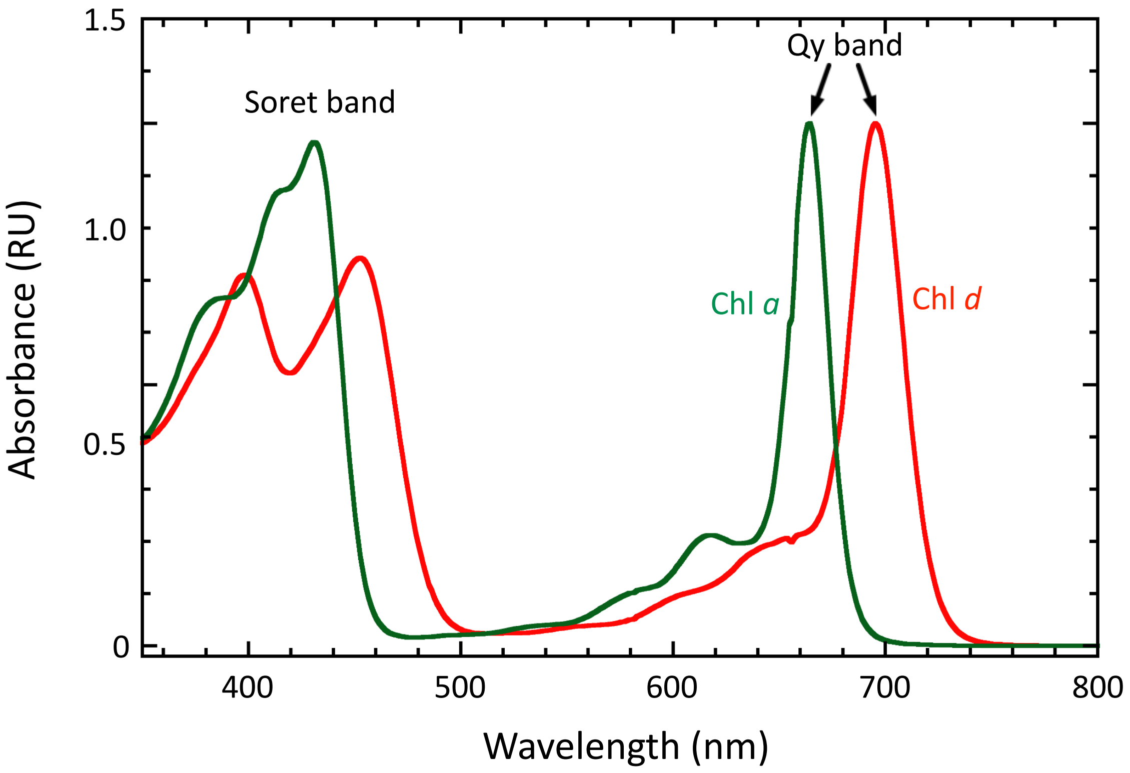

Supplementary Fig. 3: Absorption spectra of two chlorophylls.

Absorption spectrum of Chl *a* (green line) and *d* (red line) measured during HPLC analysis shown in Supplementary Fig. 19. Spectra were normalized by the peak height of the Qy band. Background noise derived from the bright line spectrum at 656.1 nm from a deuterium light source is not removed.

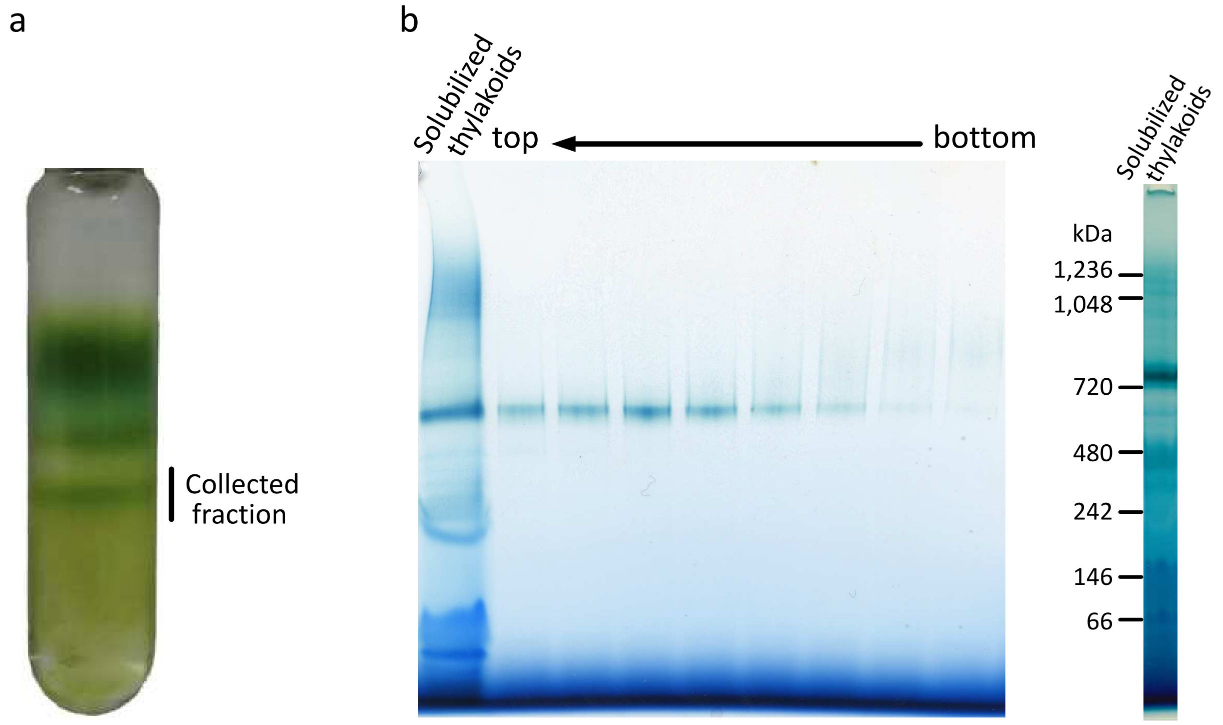

Supplementary Fig. 4: Separation of solubilized membrane protein complexes by sucrose density gradient ultracentrifugation and BN-PAGE profile of the fractions containing *A. marina* PSI trimer.

(a) Thylakoids solubilized by 1.0% UDM were fractionated by stepwise sucrose density gradient ultracentrifugation. (b) The bottom green band corresponding to the sucrose concentrations of 1.05–1.0 M was collected as PSI trimer fraction. Every equivalent to 100 μL in the sample volumes was collected from the bottom upward, and the fractions corresponding to the bottom green band were applied to BN-PAGE on linear gradient gels of 3%–12% polyacrylamide (NativePage Bis-Tris).

BN-PAGE was performed every time upon purification of PSI after sucrose density gradient centrifugation with every equivalent to 500 μL in the sample volumes, and essentially the same results were obtained. Because of the limit of sample-well number of the gel, molecular size markers were not applied to the same gel. Instead, a gel image with solubilized thylakoids and molecular size marker is presented as a reference. Source data are provided as a Source Data file.

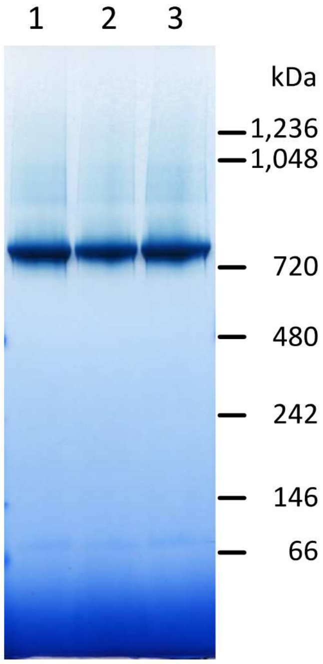

Supplementary Fig. 5. Stability of *A. marina* PSI.

Lane 1, PSI trimer stored at -80ºC after purification in the presence of 0.2% DM; lane 2, PSI trimer incubated at 0ºC under the dark for five days after purification in the presence of 0.2% DM; lane 3, PSI trimer incubated at 0ºC under the dark for five days after replacement of DM to 0.002% LMNG. PSI trimer equivalent to 3 µg Chl *d* were subjected to analysis by BN-PAGE. Positions of molecular size marker proteins are shown on the right.

After additional three months incubation of these samples at 0ºC under the dark gave essentially the same stability assessed by BN-PAGE. Source data are provided as a Source Data file.

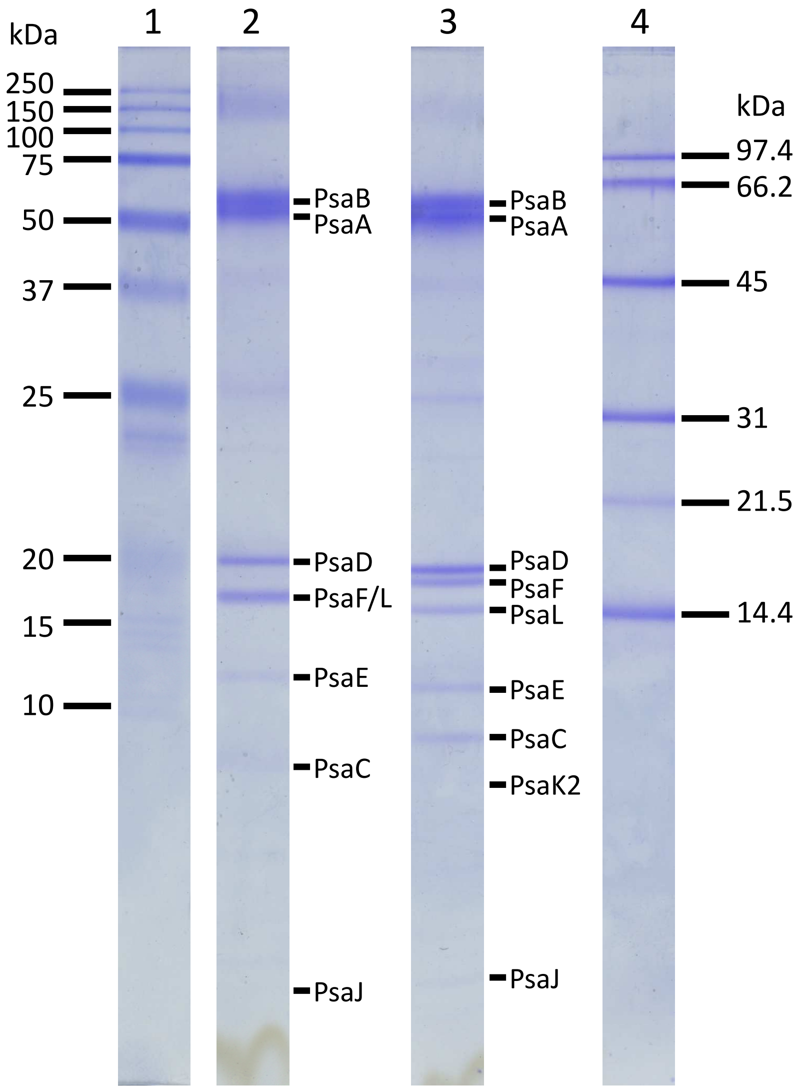

Supplementary Fig. 6: Polypeptide profile of purified PSI trimer on SDS-PAGE.

PSI trimer (lot #5 in Supplementary Table 6) equivalent to 5 µg Chl *d* was subjected to SDS-PAGE analysis with (lane 2) or without (lane 3) dithiothreitol. Molecular size marker proteins are shown in lanes 1 and 4 [lane 1, Precision Plus Protein Standards (Bio-Rad, Hercules, CA), and lane 4, LMW Marker Kit (GE Healthcare, Chicago, IL)]. The lanes were cut-out from an identical gel with the same brightness. The polypeptide identities were determined by MS analysis after in-gel digestion by trypsin (Supplementary Table 2). Polypeptide patterns essentially equivalent to lane 3 with dithiothreitol were observed for other lots listed in Supplementary Table 6. Source data are provided as a Source Data file.

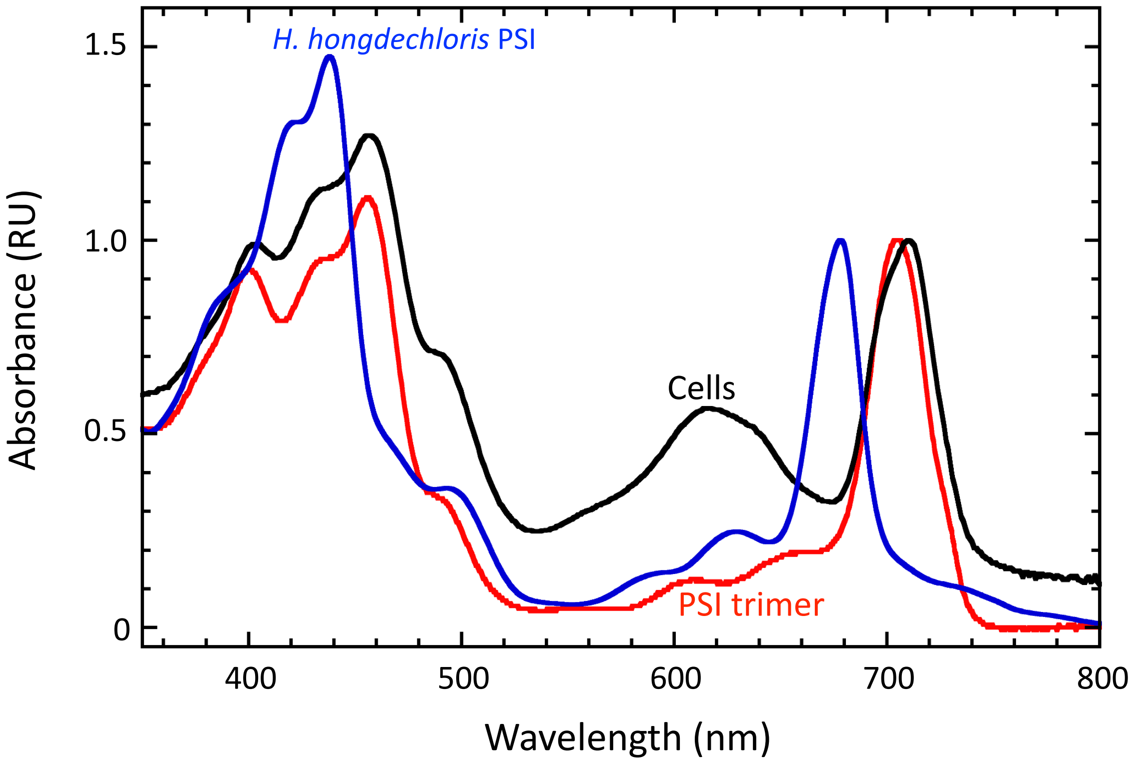

Supplementary Fig. 7: Absorption spectra of *A. marina* PSI trimer and cells.

Absorption spectrum of *A. marina* cells (black line) equivalent to 8.1 µg Chl *d* measured with an integrating sphere. Absorption spectrum of PSI trimer (red line) measured at 2.0 µg Chl *d*. Absorption spectrum of PSI isolated from cells of Chl *f*-carrying *Halomicronema hongdechloris* grown in far red light (blue line), which is a courtesy of Prof. Tatsuya Tomo, is also shown for reference. Spectra were normalized by the peak height of the Qy band.

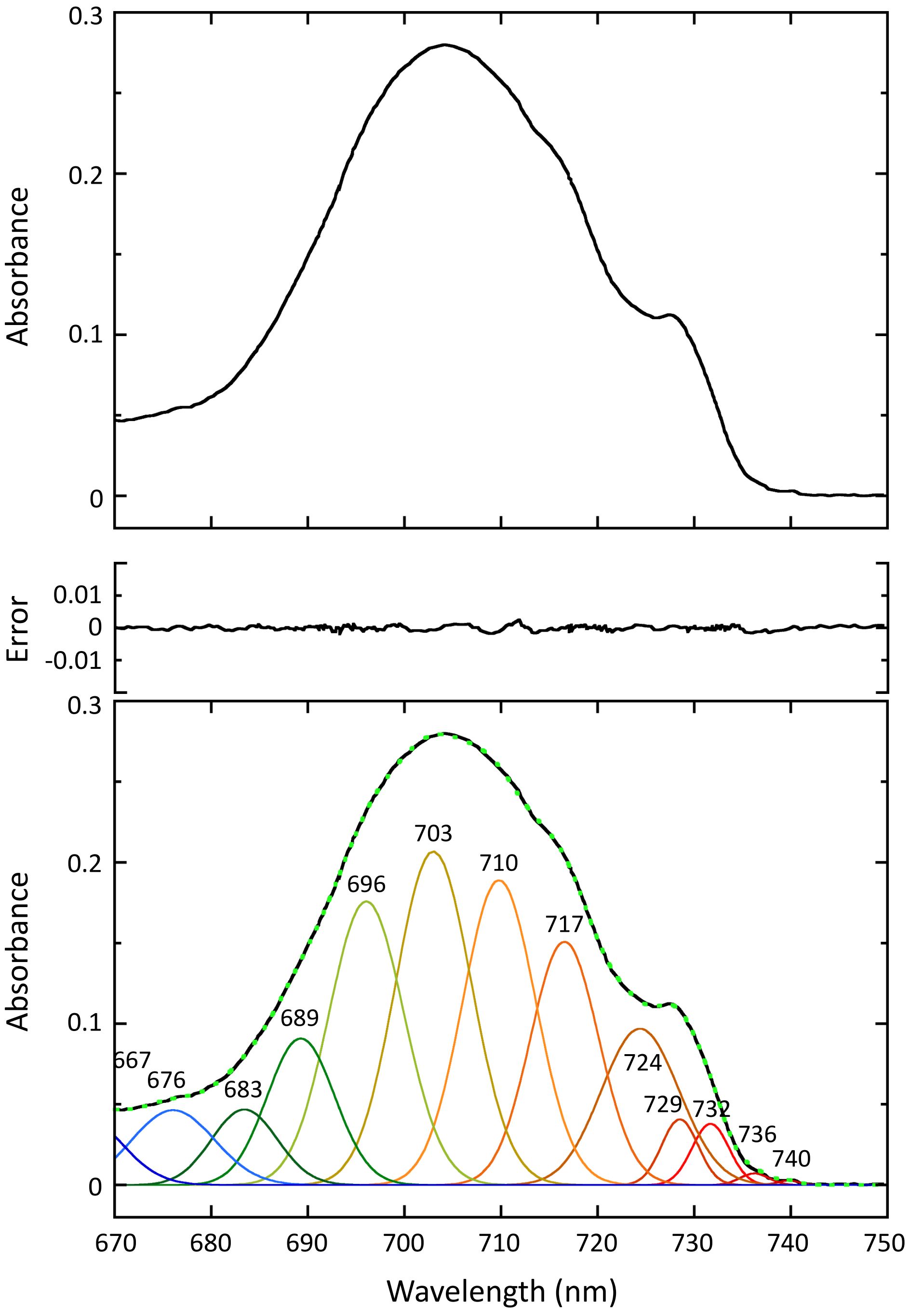

Supplementary Fig. 8: Absorption spectra of PSI trimer at 77K.

(Top) Absorption spectrum of *A. marina* cells equivalent to 10 μg Chl *d* measured using a special cuvette with a 2.0-mm light path. (Bottom) Curve fitting was performed using identical spectra converting wavelength to wavenumber using MagicPlot (ver. 2.9.3; https://magicplot.com) on MacOS. Source data are provided as a Source Data file.

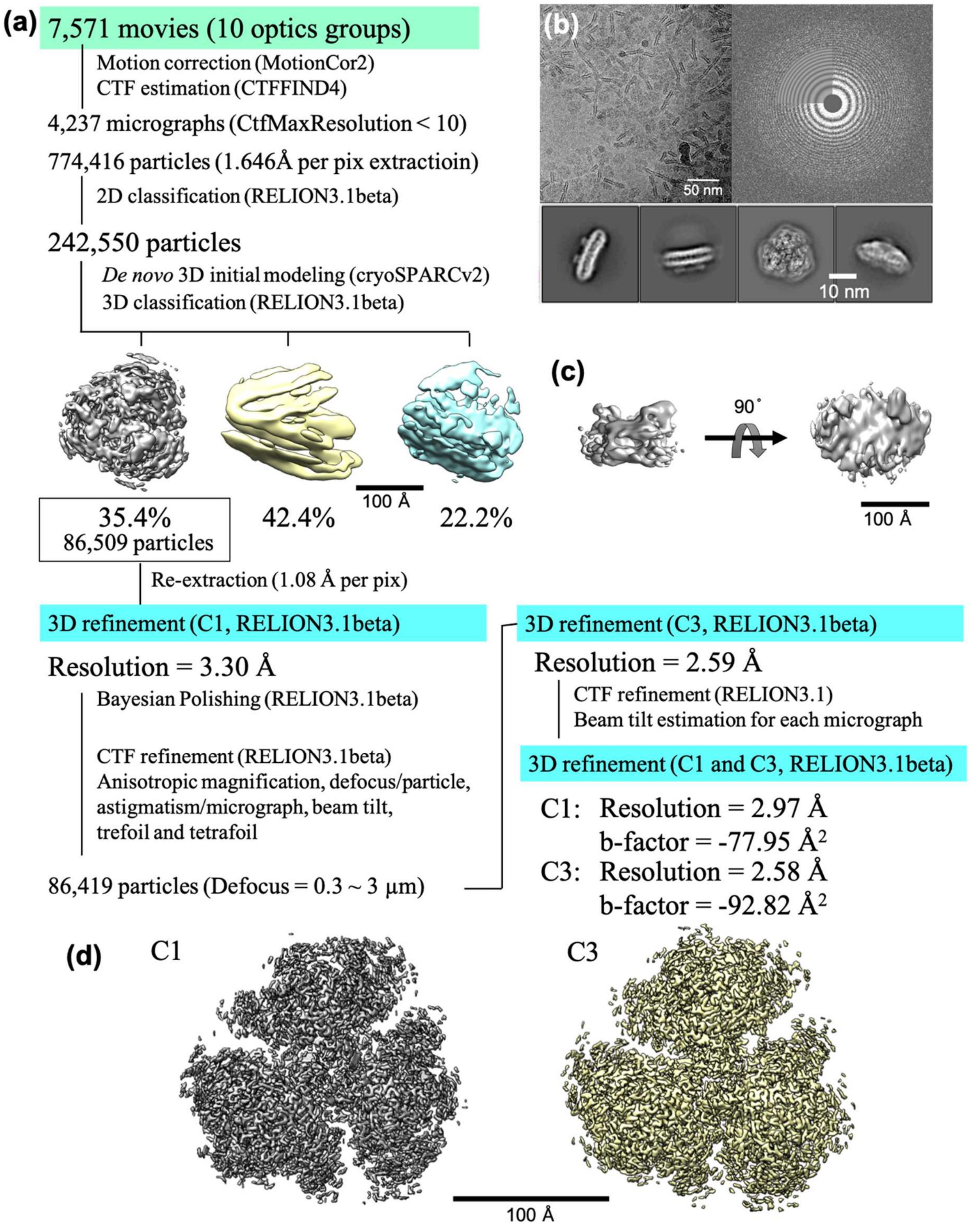

Supplementary Fig. 9: Data collection and image processing workflow of cryo-EM.

(a) Overview of the data processing workflow. (b) Representative micrograph after motion correction and class averaged images from PSI dataset. The equivalent 4,237 images were used for reconstruction. (c) Initial model of *A. marina* PSI produced using cryoSPARCv2. (d) Final map imposing symmetry of C1 and C3 reconstructed from 86,419 particles at 2.97 and 2.58 Å resolution, respectively. The resolutions were estimated based on the gold standard Fourier shell correlation (FSC) criteria, where the FSC between two independently reconstructed volumes drops to 0.143.

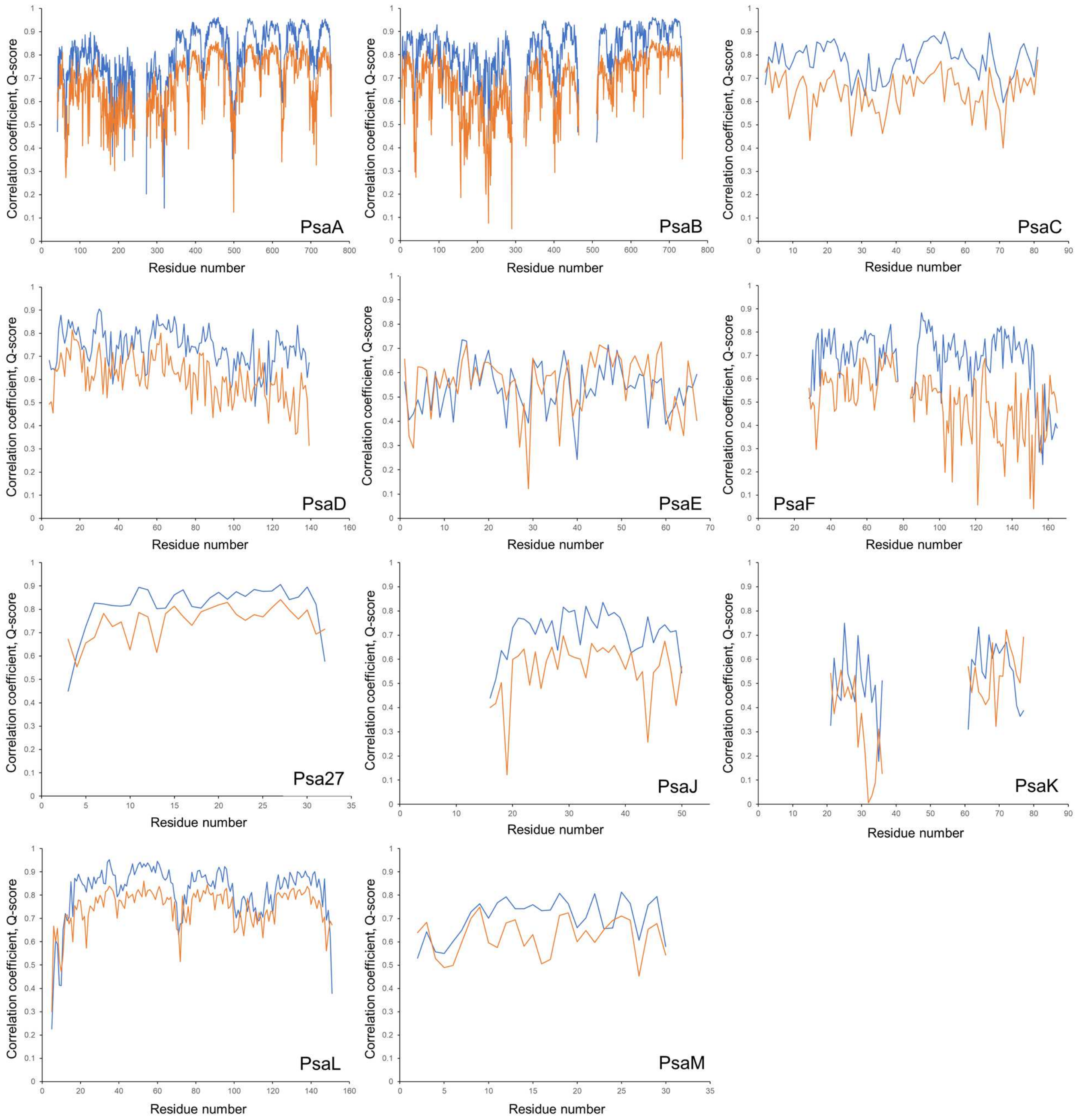

Supplementary Fig. 10: CC and Q-score values of each amino acid residue.

CC and Q-score are indicated by blue and orange lines, respectively. An area without the lines indicates that an amino acid residue was not assigned.

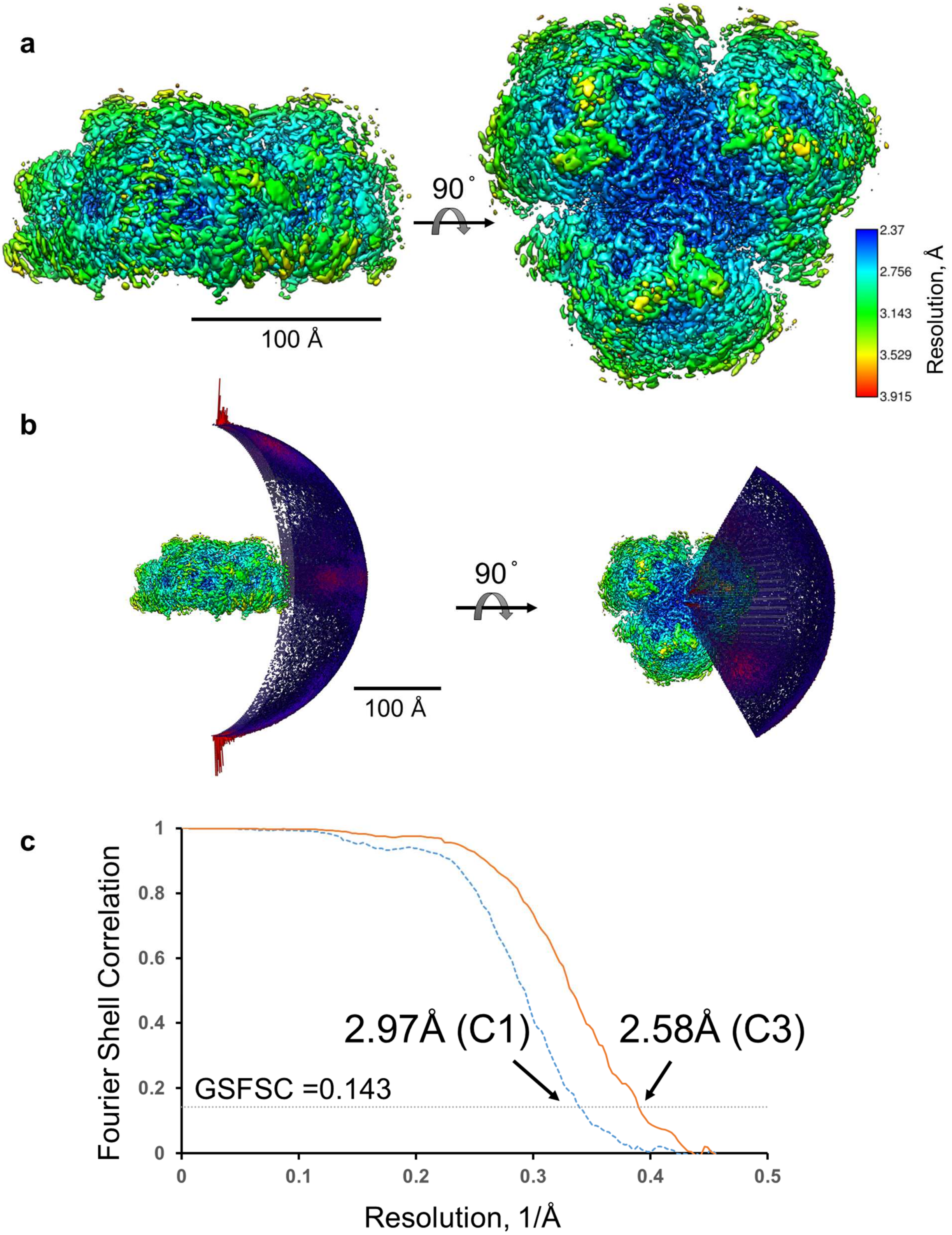

Supplementary Fig. 11: Final density map of *A. marina* PSI with C3 symmetry imposed.

(a) The cryo-EM density maps colored with estimated local resolution. (b) Euler angle distribution plot of all particles used for the final map. (c) Gold standard FSC curves between two half-maps independently refined in the reconstruction.

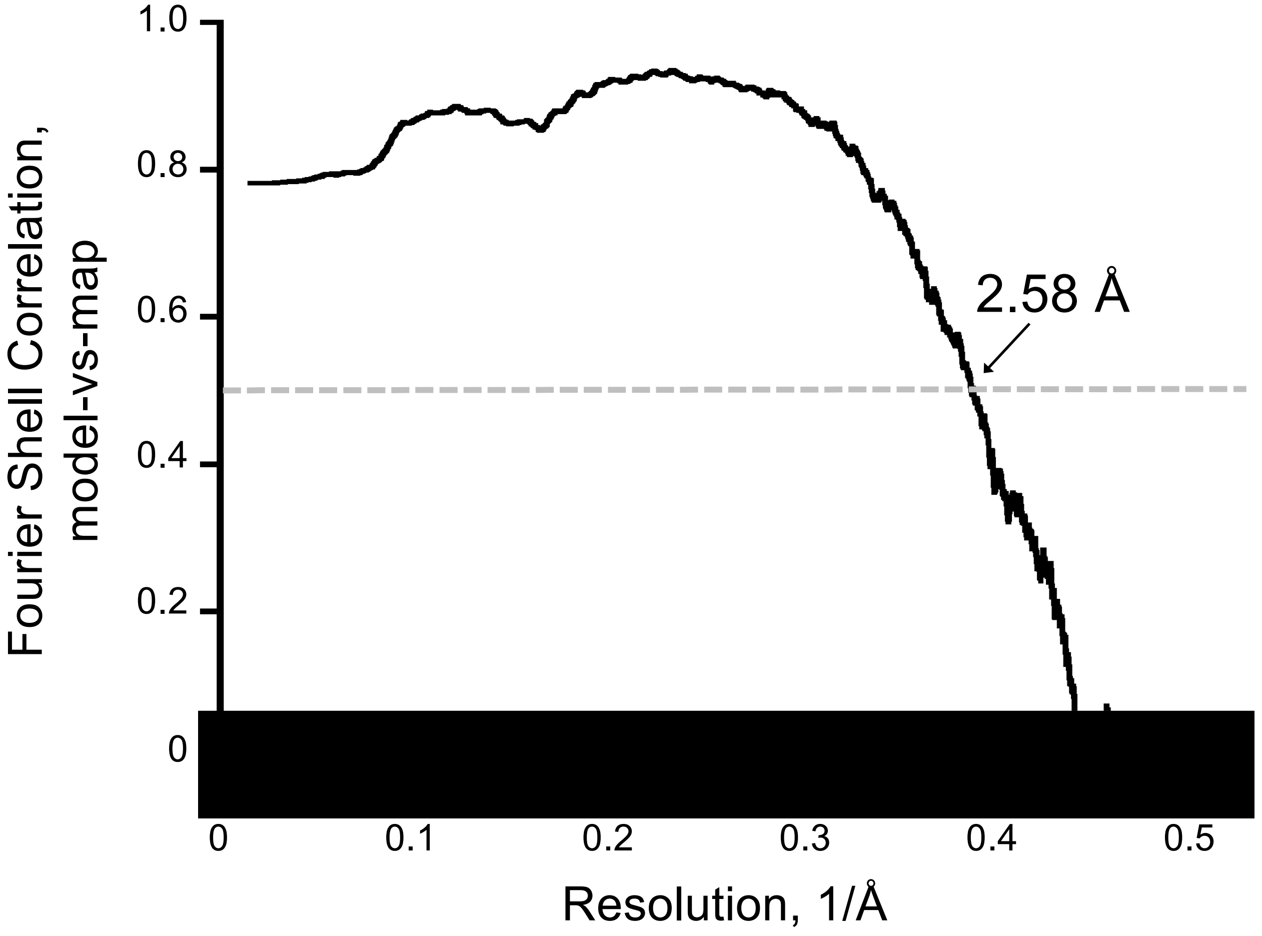

Supplementary Fig. 12: FSC curve calculated between the cryo-EM map and the refined structure model of *A. marina* PSI.

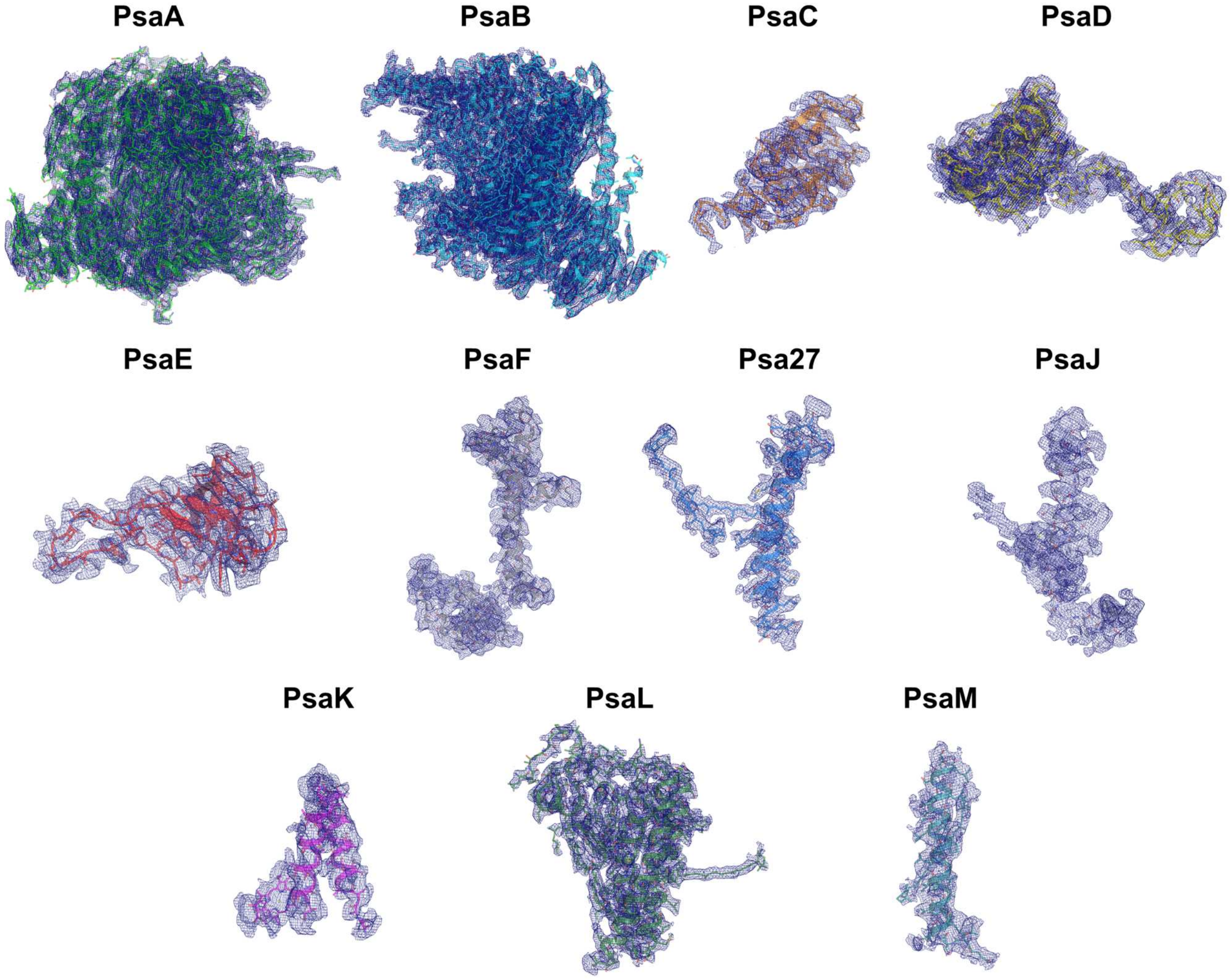

Supplementary Fig. 13: Cryo-EM density map of each protein subunit of *A. marina* PSI.
Each cryo-EM density map is shown in a mesh representation at 1.0 sigma contour level.

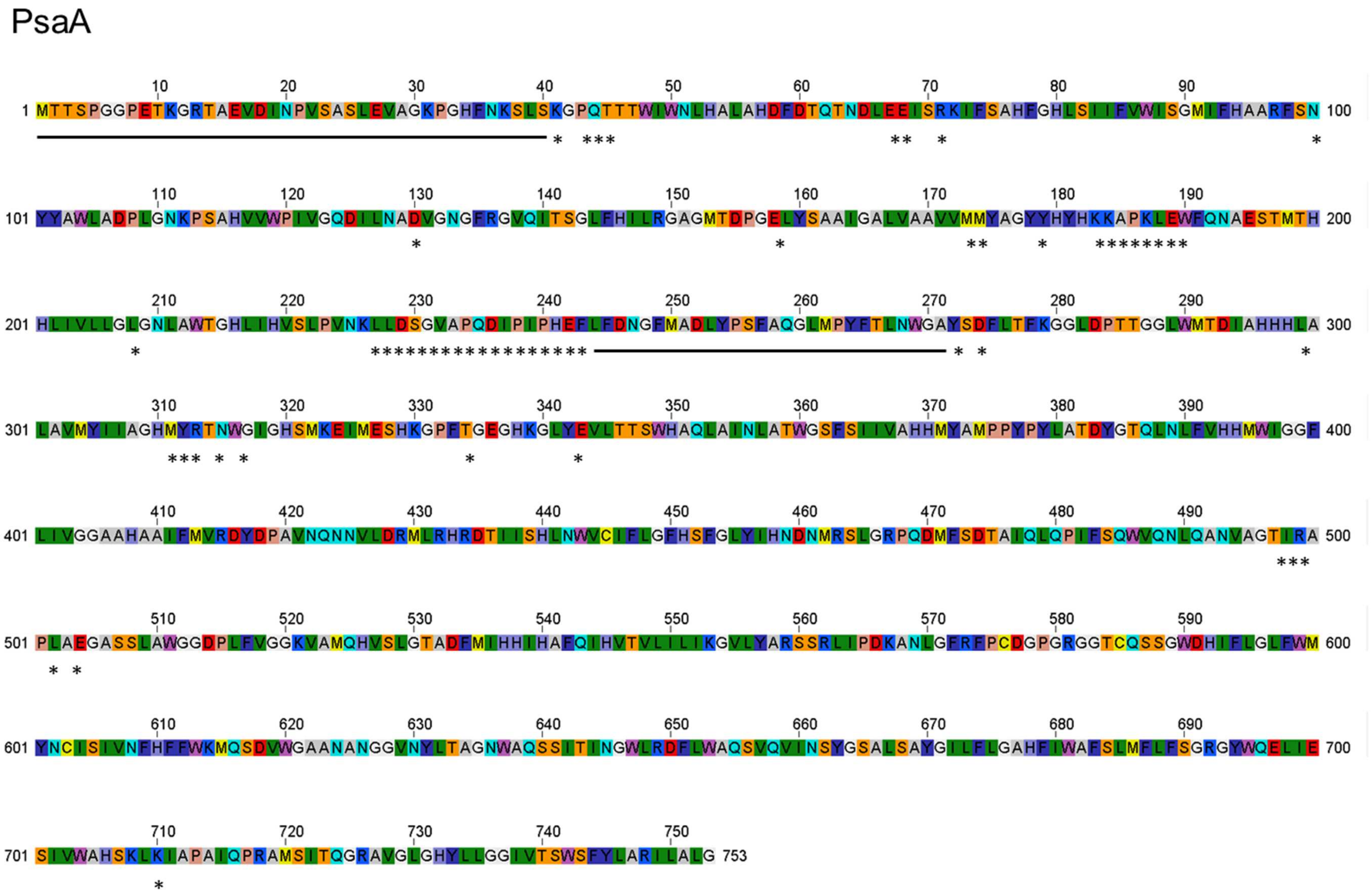

Supplementary Fig. 14-a: Assignment of the amino acid residues in PsaA.

"-" is a non-assigned amino acid residue and "*" is assigned as alanine because of low cryo-EM map quality.

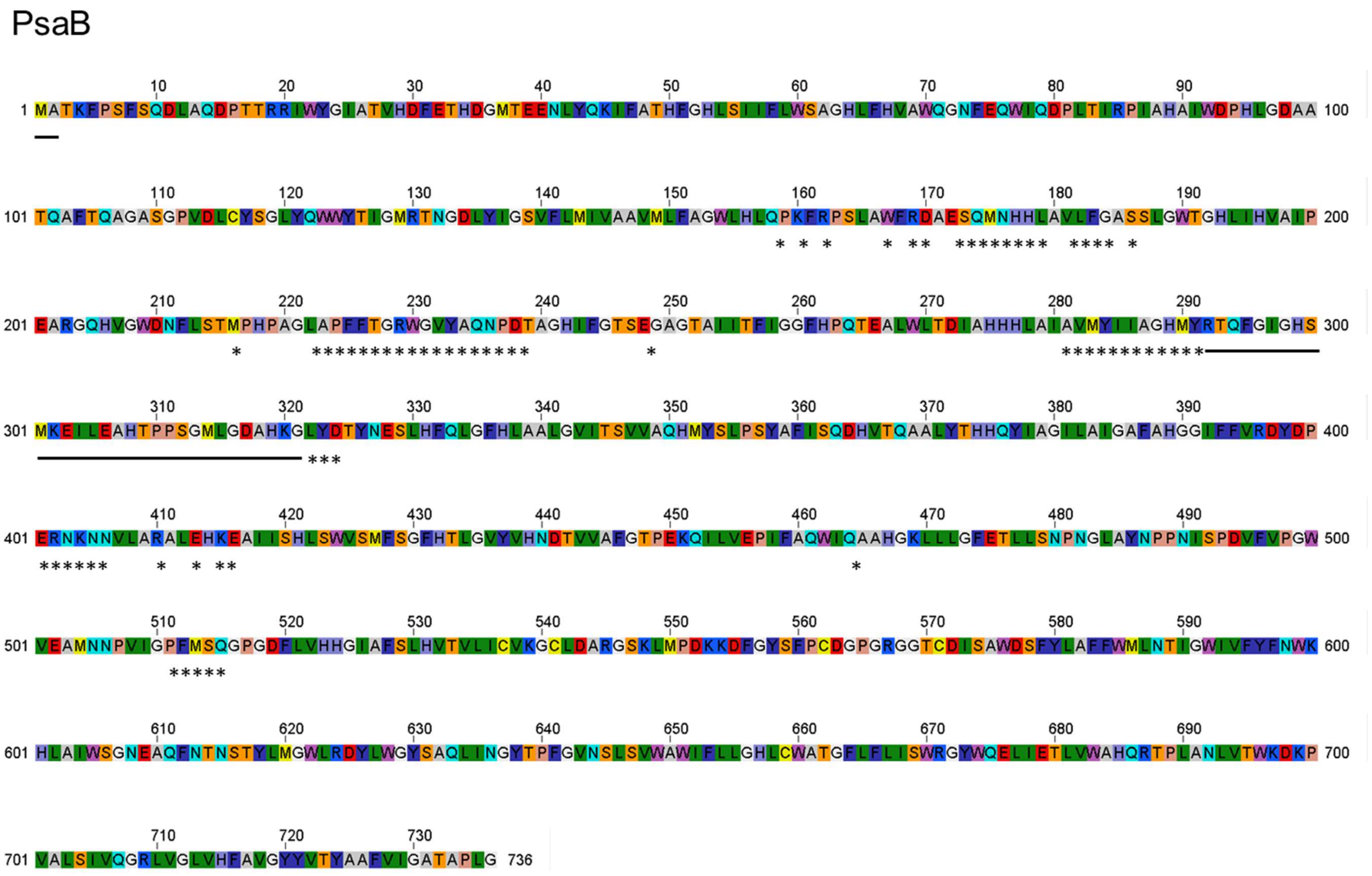

Supplementary Fig. 14-b: Assignment of the amino acid residues in PsaB.

"-" is a non-assigned amino acid residue and "*" is assigned as alanine because of low cryo-EM map quality.

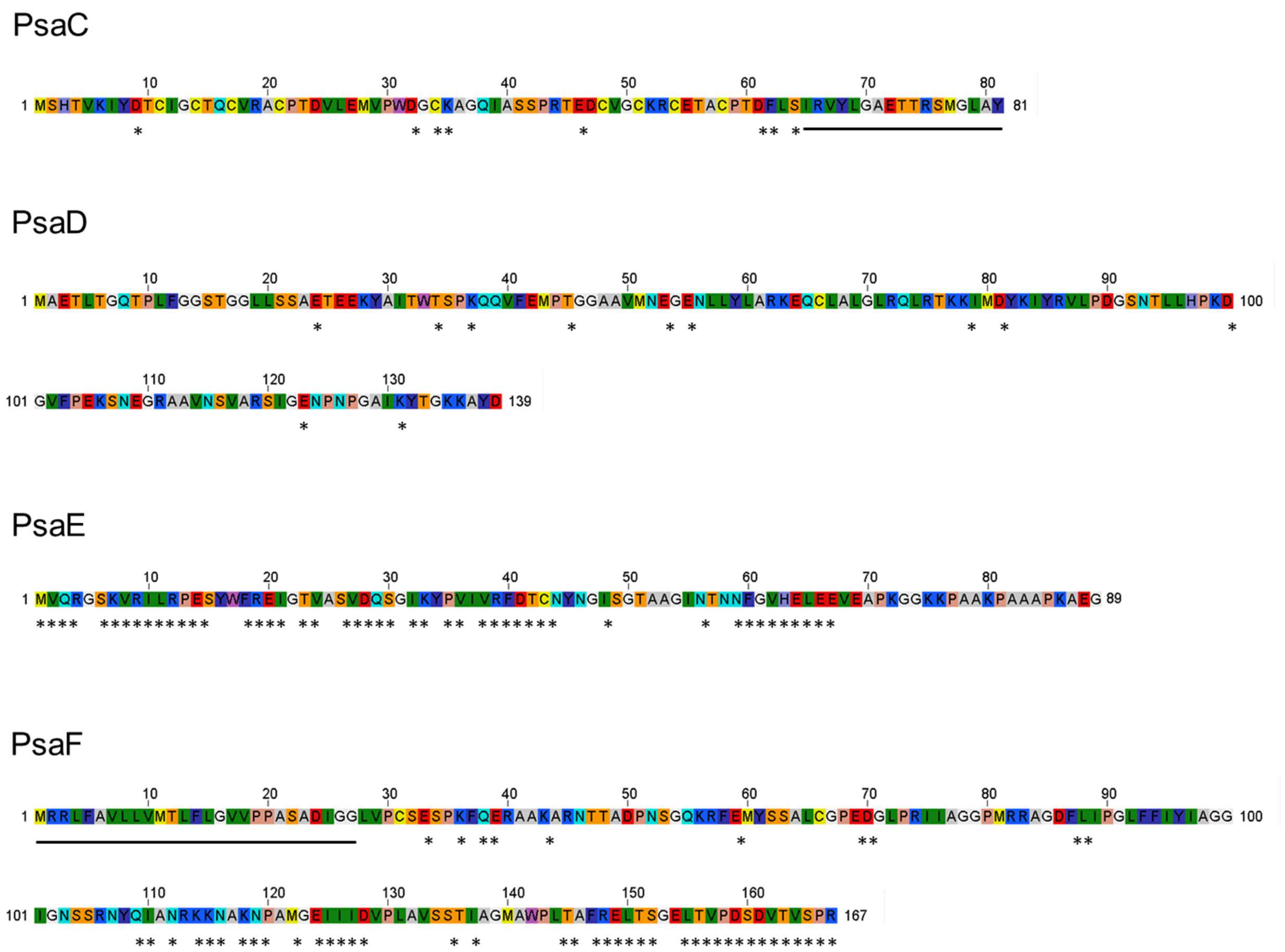

Supplementary Fig. 14-c: Assignment of the amino acid residues in PsaC, PsaD, PsaE and PsaF.

"-" is a non-assigned amino acid residue and "*" is assigned as alanine because of low cryo-EM map quality.

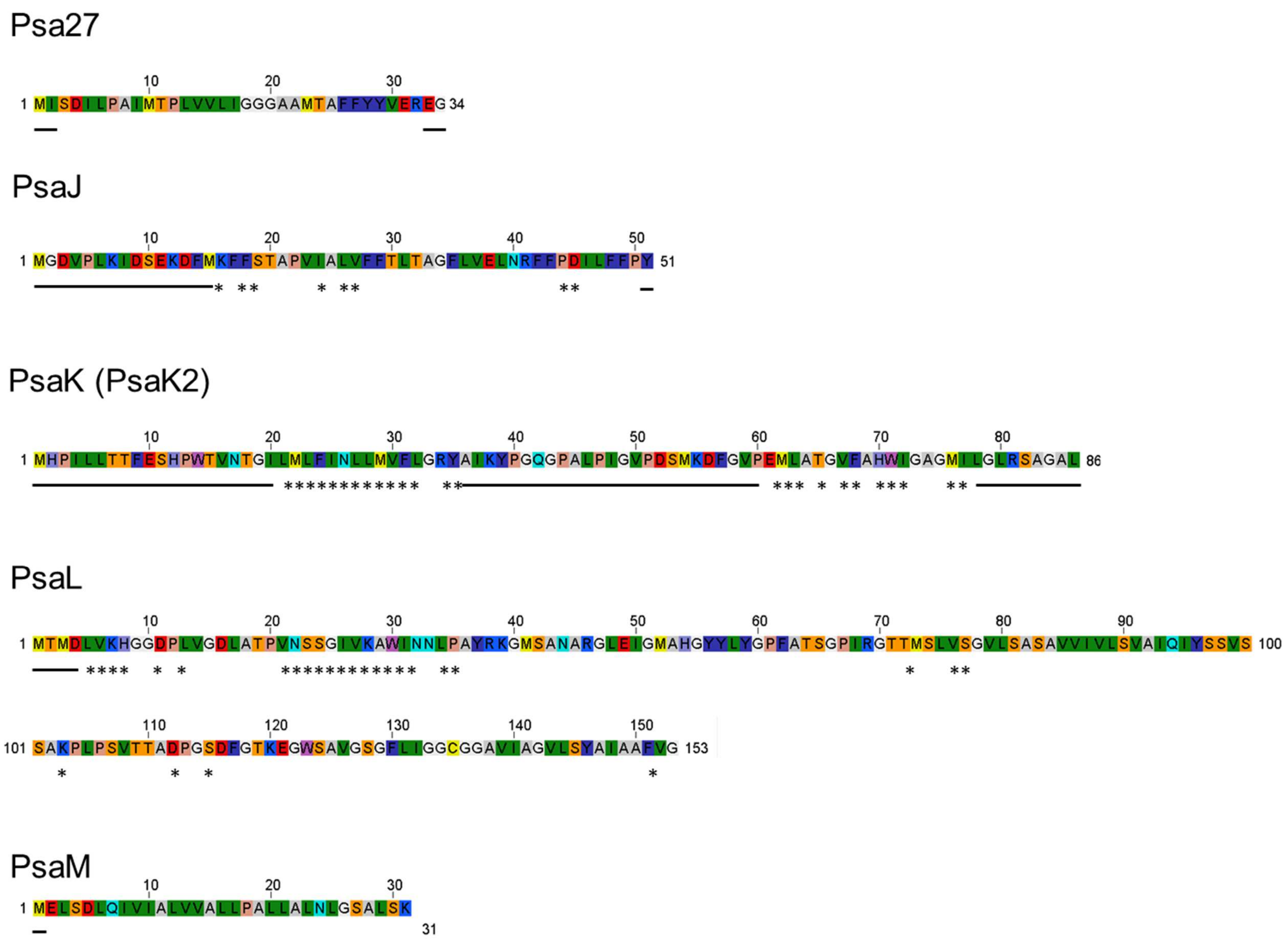

Supplementary Fig. 14-d: Assignment of the amino acid residues in Psa27, PsaJ, PsaK, PsaL and PsaM.

“-” is a non-assigned amino acid residue and “*” is assigned as alanine because of low cryo-EM map quality.

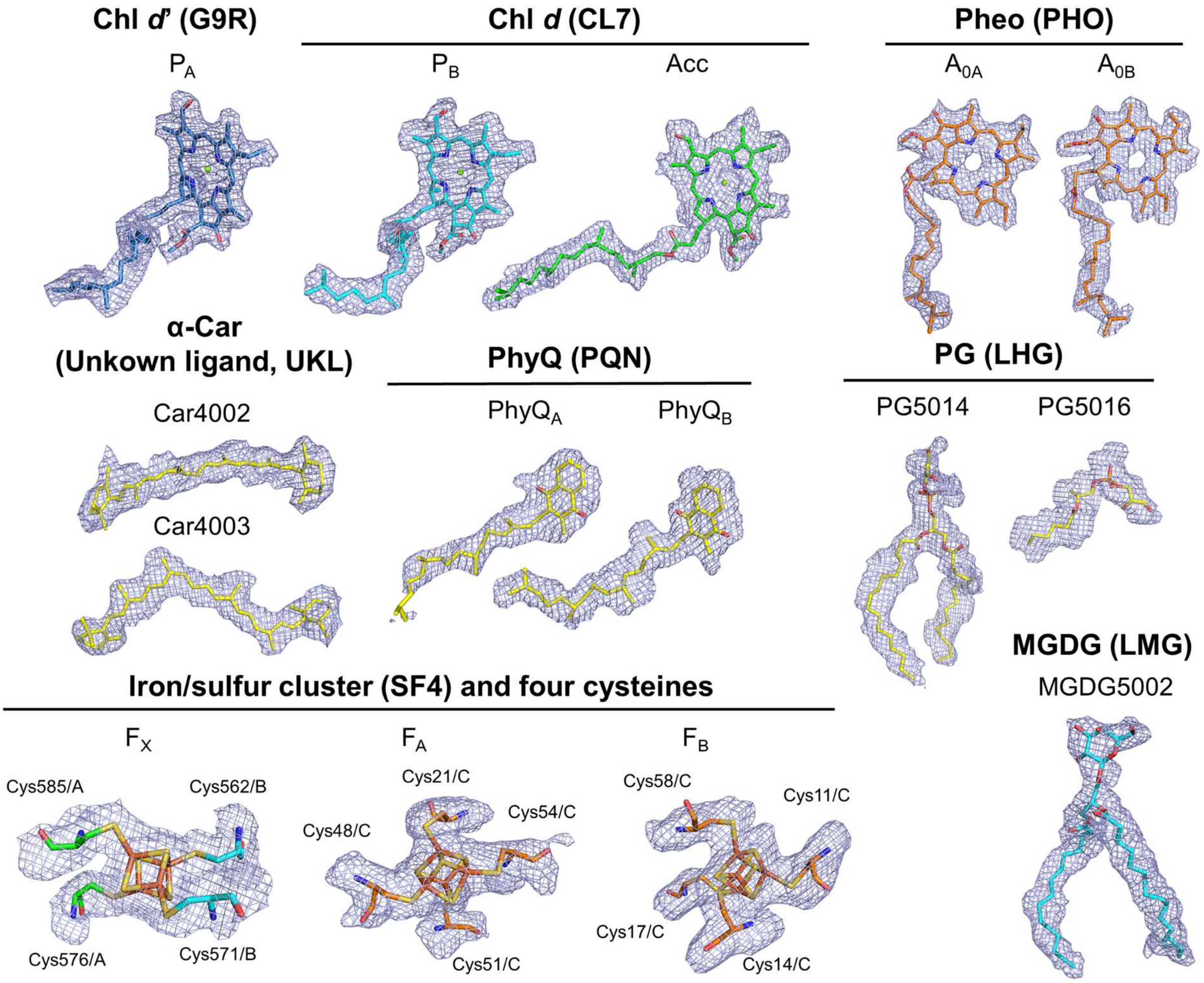

Supplementary Fig. 15: Cryo-EM density map for each cofactor.

Each cryo-EM density map is shown in a mesh representation at 1.0 sigma contour level. Each ligand ID is indicated in parentheses. The numbers for α-Car, PG, and MGDG indicate the respective residue numbers. Chlorophyll *d*, Chl *d*; chlorophyll *d'*, Chl *d'*; pheophythin, Pheo; α-carotene, α-Car; phosphatidylglycerol, PG; monogalactosyl diacylglycerol, MGDG.

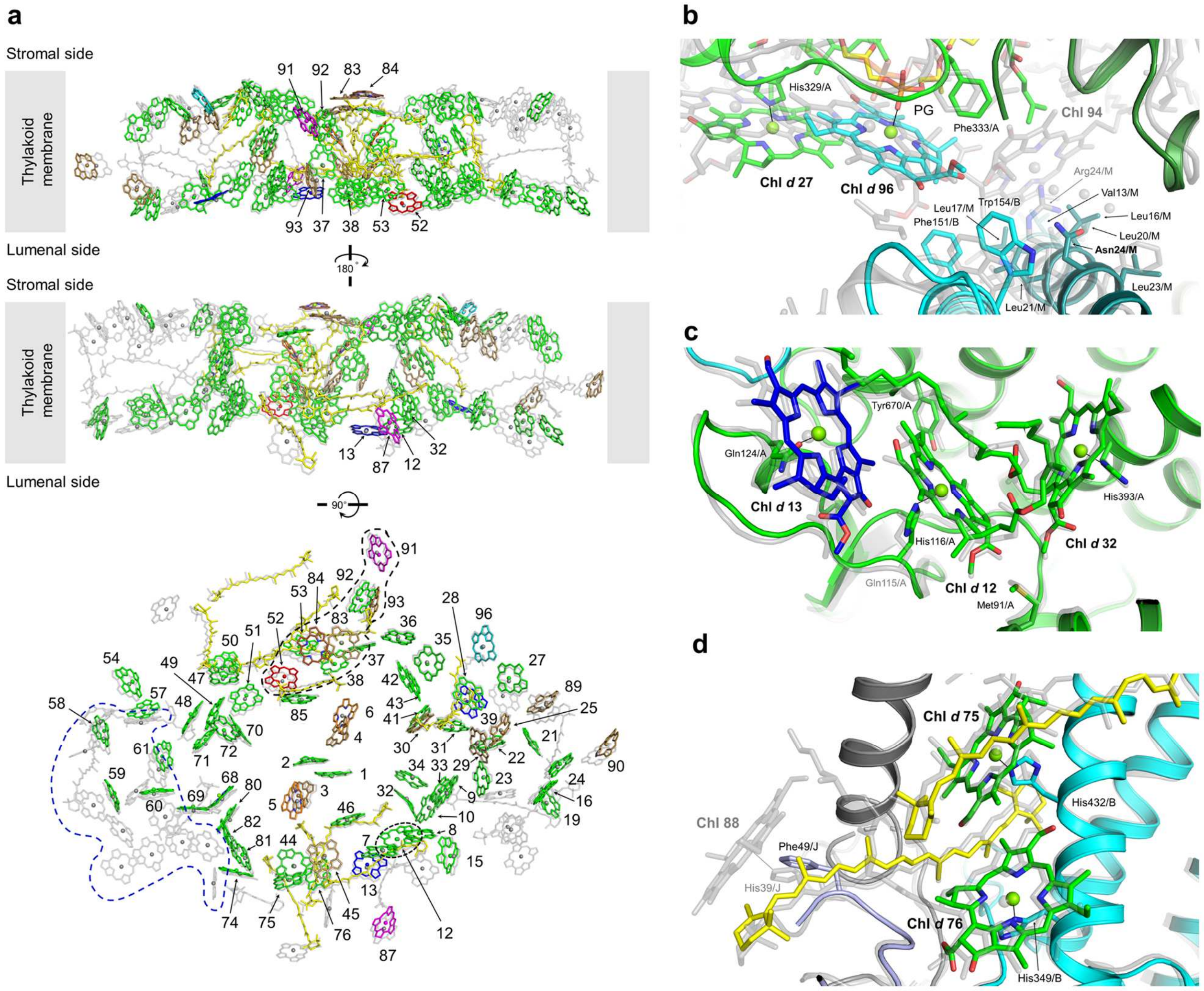

Supplementary Fig. 16: Arrangement of pigments in PSI.

(a) Superposition of pigments of *A. marina* PSI with those from *T. elongatus* (transparent grey). The lower figure is drawn as in Fig. 2C but rotated 90° counterclockwise. Pigments of *A. marina* are colored according to the amino acid residue that is their ligand: green, His; brown, probable water molecule; red, Asp; blue, Gln; magenta, Glu; cyan, phosphatidylglycerol. The black-dashed line shows a region where the arrangement of pigments differs from those in *T. elongatus* PSI because of different corresponding amino acids in subunits Psa27 (PsaI) and PsaL. The blue-dashed line shows a disordered region of *A. marina* PsaB. (b) (c) (d) Pigment–pigment interaction and the surrounding structures. Amino acid residues and pigments of *T. elongatus* are shown in transparent images. Solid lines show coordination bonds and dashed lines show Mg^{2+}–Mg^{2+} distances between Chls.

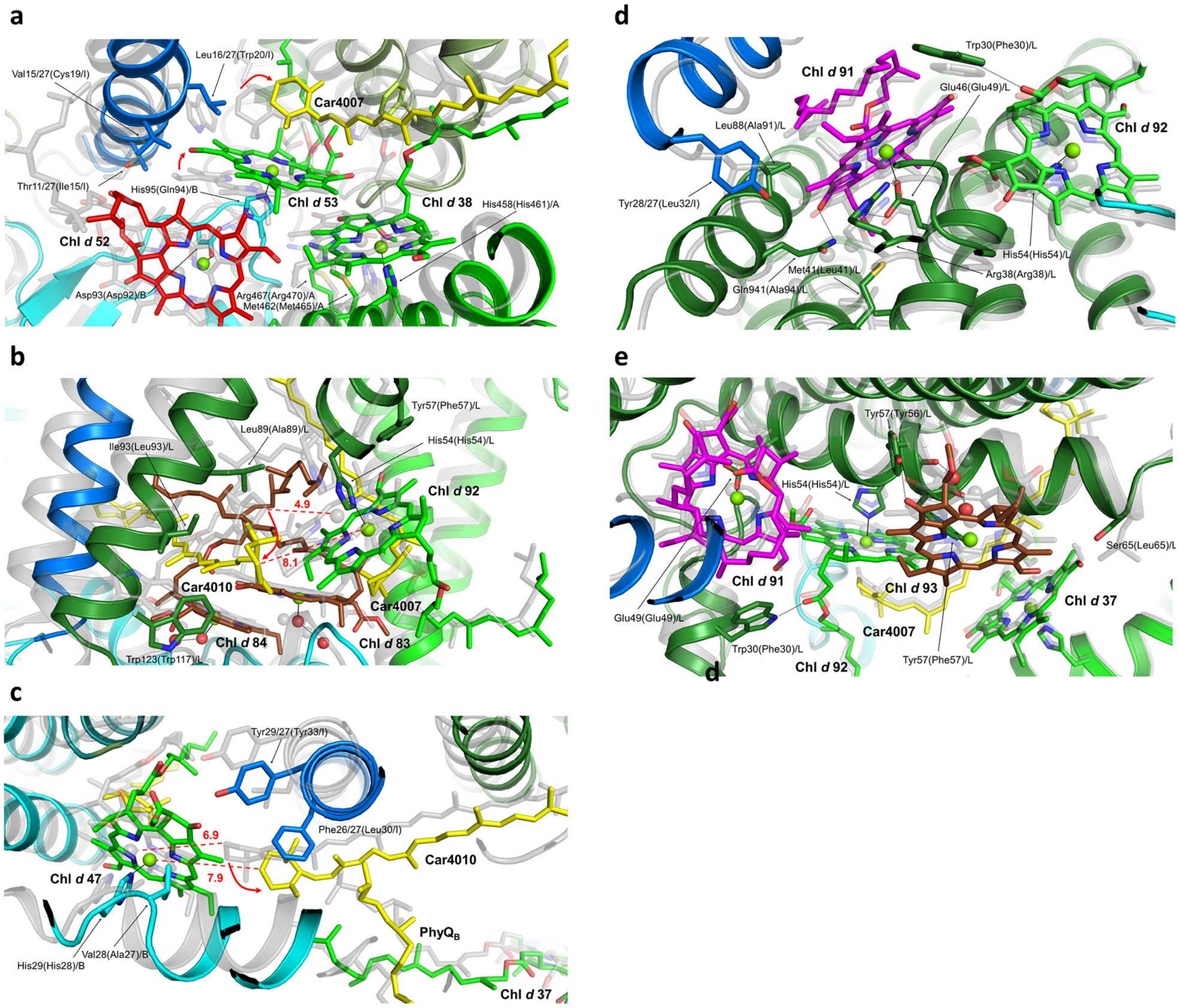

Supplementary Fig. 17: Detailed arrangement of some pigments in *A. marina* PSI that are different from those in *T. elongatus* PSI (within the black-dashed line in Fig. 17a).

Non-transparent, *A. marina*; transparent grey, *T. elongatus*. (a) Arrangement of Chl *d* 38, 52, and 53 and Car4007 and their surrounding environments. (b) and (c) Arrangement of Car4010. Distances between rings of Ca4010 and Mg^{2+} ions chelated in Chl *d* or *a* are shown in red. (d) and (e) Amino acid environments around Chl *d* 37, 91, 92 and 93 located near the center of the PSI trimer.

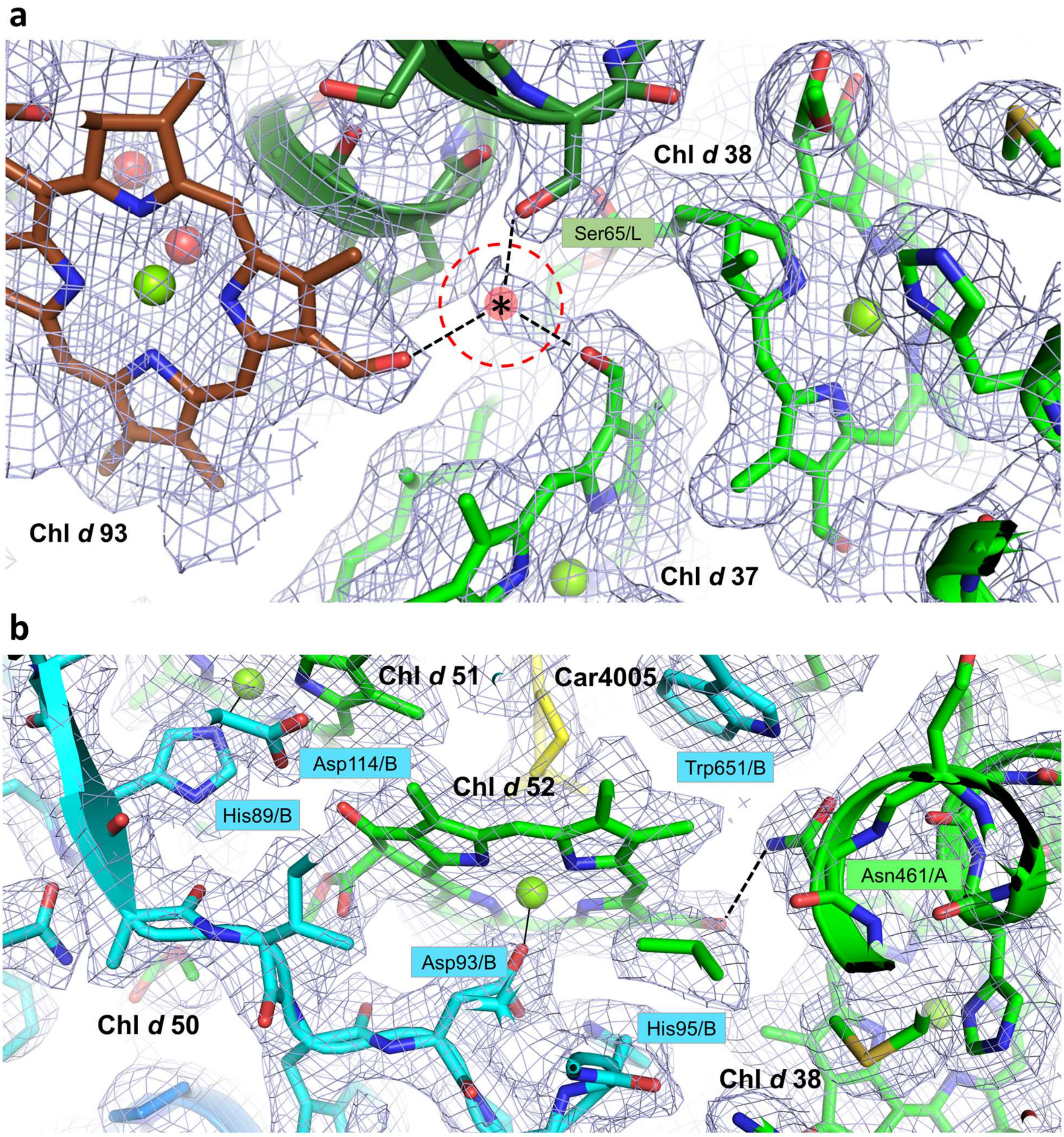

Supplementary Fig. 18: Representative examples of hydrogen bonds with the C3-formyl group of Chl *d*.

(a) Cryo-EM density map around Chls (Chl *d* 37, Chls *d* 38, and Chl *d* 93) of *A. marina* PSI shown in a mesh representation at 4.0 sigma contour level. One water molecule (asterisk) probably forms hydrogen bonds with neighboring Chls (Chl *d* 37 and Chl *d* 93) and Ser65/L. However, this water molecule was not assigned in the refined structure model because these were shorter than the typical hydrogen bond distance. (b) Cryo-EM density map around Chl *d* 52 of *A. marina* PSI shown in a mesh representation at 3.0 sigma contour level. The C3-formyl group of Chl *d* 52 forms hydrogen bond (dashed line) with Asn461/A.

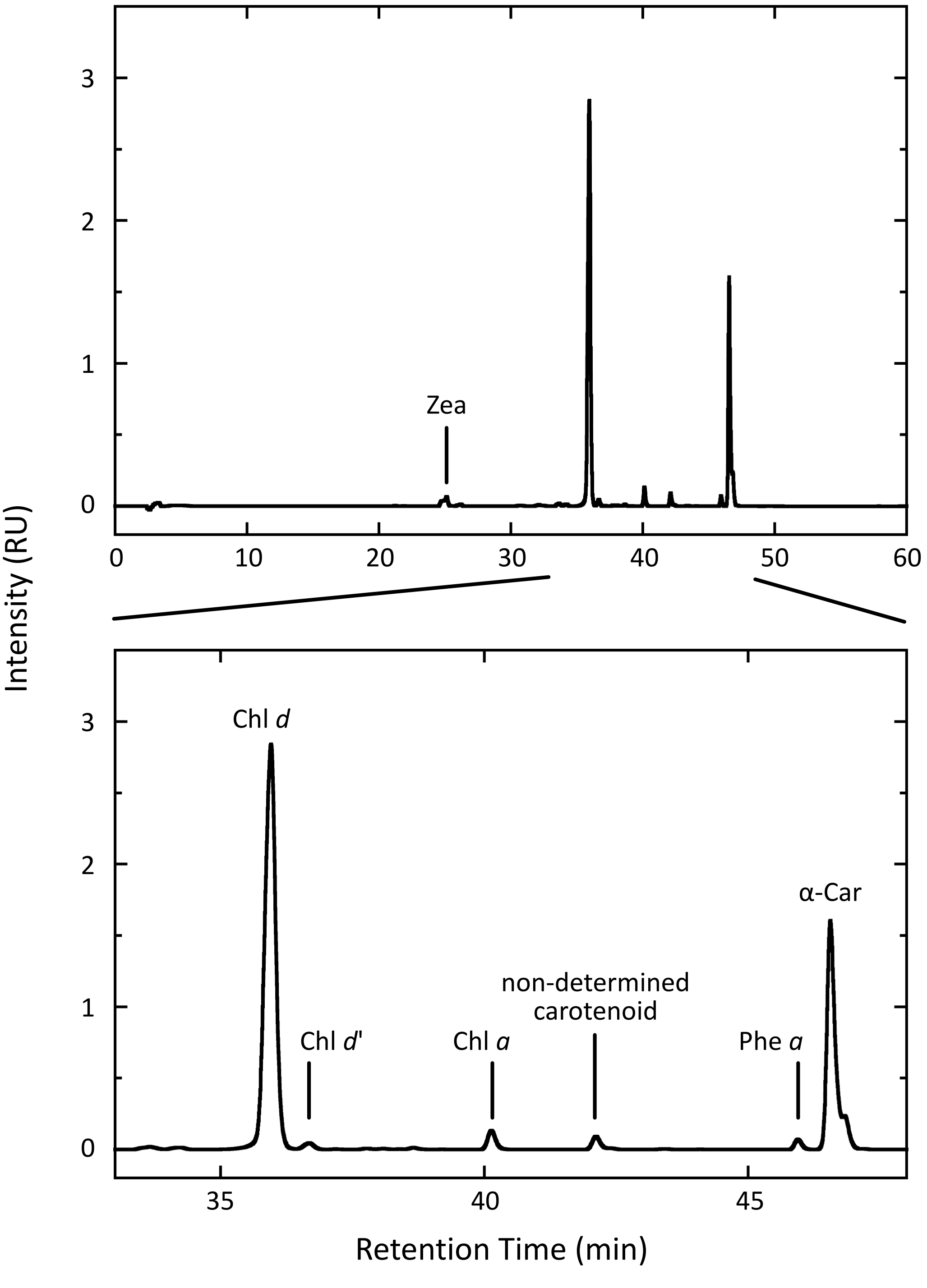

Supplementary Fig. 19: Typical HPLC chromatogram.

Pigments extracted from *A. marina* PSI trimer were applied to HPLC analysis to determine pigment composition. Chromatogram at 430 nm. α-Car, α-carotene; Chl, chlorophyll; Phe, pheophytin; Zea, zeaxanthin.

```
>ENA|ABW27465|ABW27465.1 Acaryochloris marina MBIC11017
photosystem I core protein PsaA
ATGACAACTAGCCCAGGTGGGCCAGAGACAAAAGGCAGAACAGCTGAAGTTGACATCAAC
CCAGTTAGCGCTTCTTTAGAAGTCGCGGGTAAGCCGGGTCACTTTAATAAAAGTCTGTCG
AAAGGTCCCCAAACCACCACTTGGATTTGGAACCTACATGCTCTAGCCCATGATTTTGAT
ACACAAACAAACGACCTAGAAGAAATTTCCCGCAAAATTTTCAGTGCCCATTTTGGACAC
TTATCCATCATTTTTGTATGGATCAGCGGGATGATCTTCCATGCTGCTCGTTTTTCTAAC
TACTACGCTTGGTTAGCCGATCCGCTCGGCAACAAACCCAGTGCTCACGTAGTTTGGCCC
ATTGTTGGCCAAGATATTTTAAATGCAGATGTTGGTAATGGATTCCGCGGAGTCCAAATT
ACCTCTGGCCTTTTCCATATTTTACGCGGGGCTGGAATGACTGACCCCGGTGAACTTTAT
TCAGCAGCCATTGGTGCCCTTGTTGCAGCGGTTGTAATGATGTACGCCGGGTATTATCAC
TACCACAAGAAAGCACCTAAATTGGAGTGGTTCCAAAACGCCGAATCAACGATGACCCAC
CATCTCATCGTTCTTTTAGGGCTTGGGAACCTTGCCTGGACAGGTCACCTTATCCATGTT
TCTCTGCCAGTCAATAAGCTTCTTGATTCTGGTGTAGCCCCACAAGATATACCAATCCCC
CATGAATTTCTTTTTGATAATGGATTTATGGCGGATTTATATCCCAGCTTTGCTCAGGGA
TTAATGCCTTACTTCACCCTAAATTGGGGTGCTTATTCTGACTTCCTTACCTTCAAAGGA
GGGCTTGACCCAACAACGGGTGGCCTATGGATGACAGATATAGCCCATCACCATTTGGCA
TTGGCAGTAATGTACATCATTGCTGGTCATATGTACCGAACCAACTGGGGTATTGGGCAC
AGTATGAAGGAAATCATGGAATCTCATAAAGGTCCCTTTACTGGCGAAGGCCATAAAGGT
CTATATGAGGTGCTGACAACTTCTTGGCATGCCCAGCTAGCAATTAACCTAGCCACATGG
GGTTCTTTCAGCATCATTGTTGCCCACCACATGTACGCAATGCCTCCTTATCCTTACTTG
GCAACAGATTACGGCACGCAGCTGAATCTGTTCGTCCATCATATGTGGATTGGAGGTTTC
TTGATTGTTGGTGGTGCTGCCCACGCAGCTATTTTCATGGTTCGGGATTACGATCCAGCT
GTGAACCAAAACAATGTTCTTGATCGGATGCTTCGTCACCGAGATACGATCATTTCCCAT
CTAAACTGGGTCTGTATTTTCCTTGGGTTCCATTCTTTTGGCTTGTATATCCATAACGAC
AATATGCGTTCTTTGGGTCGGCCTCAAGATATGTTCTCCGACACTGCTATCCAACTGCAA
CCTATTTTTTCTCAATGGGTTCAGAACTTACAAGCAAACGTTGCTGGAACAATTCGGGCT
CCCTTGGCAGAAGGTGCATCAAGCTTAGCTTGGGGTGGCGATCCTTTGTTTGTTGGCGGA
AAAGTTGCAATGCAACATGTTTCCTTAGGAACCGCCGATTTCATGATCCACCACATTCAC
GCCTTCCAGATTCACGTTACTGTTCTCATCCTAATCAAGGGTGTTCTCTACGCTCGTAGC
TCTCGTCTAATTCCAGACAAAGCTAACTTGGGCTTCAGATTCCCTTGCGACGGACCAGGT
CGGGGTGGTACTTGCCAATCTTCTGGTTGGGACCATATCTTCTTGGGTCTGTTCTGGATG
TACAACTGCATCTCAATTGTCAATTTCCACTTCT TCTGGAAAATGCAGTCGGATGTTTGG
GGTGCCGCAAATGCTAATGGCGGCGTTAATTACCTAACAGCTGGCAACTGGGCACAGTCT
TCAATCACTATTAATGGTTGGTTGCGAGATTTCTTATGGGCCCAATCGGTTCAGGTGATT
AACTCCTATGGTTCTGCCCTATCTGCCTACGGAATTTTATTCCTAGGTGCCCACTTCATC
TGGGCTTTCAGCCTGATGTTCTTGTTCAGTGGTCGTGGCTATTGGCAAGAGCTGATCGAG
TCTATTGTTTGGGCTCACAGCAAACTAAAGATTGCTCCAGCCATTCAGCCACGCGCTATG
AGTATTACTCAAGGTCGTGCAGTTGGACTGGGCCATTACCTCCTAGGTGGAATTGTGACC
TCTTGGTCATTCTACCTAGCTCGAATTCT CGCATTAGGATAG
```

```
MTTSPGGPETKGRTAEVDINPVSASLEVAGKPGHFNKSLSKGPQTTTWIWNLHALAHDFD
TQTNDLEEISRKIFSAHFGHLSIIFVWISGMIFHAARFSNYYAWLADPLGNKPSAHVVWP
IVGQDILNADVGNGFRGVQITSGLFHILRGAGMTDPGELYSAAIGALVAAVVMMYAGYYH
YHKKAPKLEWFQNAESTMTHHLIVLLGLGNLAWTGHLIHVSLPVNKLLDSGVAPQDIPIP
HEFLFDNGFMADLYPSFAQGLMPYFTLNWGAYSDFLTFKGGLDPTTGGLWMTDIAHHHLA
LAVMYIIAGHMYRTNWGIGHSMKEIMESHKGPFTGEGHKGLYEVLTTSWHAQLAINLATW
GSFSIIVAHHMYAMPPYPYLATDYGTQLNLFVHHMWIGGFLIVGGAAHAAIFMVRDYDPA
VNQNNVLDRMLRHRDTIISHLNWVCIFLGFHSFGLYIHNDNMRSLGRPQDMFSDTAIQLQ
PIFSQWVQNLQANVAGTIRAPLAEGASSLAWGGDPLFVGGKVAMQHVSLGTADFMIHHIH
AFQIHVTVLILIKGVLYARSSRLIPDKANLGFRFPCDGPGRGGTCQSSGWDHIFLGLFWM
YNCISIVNFHFFWKMQSDVWGAANANGGVNYLTAGNWAQSSITINGWLRDFLWAQSVQVI
NSYGSALSAYGILFLGAHFIWAFSLMFLFSGRGYWQELIESIVWAHSKLKIAPAIQPRAM
SITQGRAVGLGHYLLGGIVTSWSFYLARI LALG*
```

Supplementary Fig. 20-a: Assessment of the accuracy of DNA sequence of *psaA* in the database. The accuracy of the DNA sequence (accession number ABW27465.1) of the blue-colored region in the upper panel was confirmed. The corresponding amino acid sequence is shown in the lower panel. The codon and residue corresponding to the ligand of Acc in *A. marina* PSI are shown in magenta. DNA sequencing was performed for forward- and reverse-reading twice, respectively, yielding the same sequence as that of accession number ABW27465.1.

```
>ENA|ABW27466|ABW27466.1 Acaryochloris marina MBIC11017
photosystem I core protein PsaB
ATGGCTACTAAATTTCCTAGTTTTAGCCAAGACCTTGCCCAAGATCCAACAACACGTCGG
ATCTGGTACGGAATTGCCACAGTTCATGATTTTGAGACTCATGACGGAATGACGGAGGAA
AATCTTTATCAAAAGATTTTCGCGACTCACTTCGGTCATCTCTCTATTATCTTTCTATGG
TCTGCTGGCCATCTTTTCCATGTCGCCTGGCAAGGCAACTTTGAACAGTGGATCCAAGAT
CCACTAACCATCCGTCCCATCGCCCATGCGATTTGGGACCCCCATTTGGGTGATGCTGCA
ACTCAGGCGTTCACCCAAGCTGGCGCTTCTGGTCCAGTTGACCTTTGTTATTCTGGCCTC
TACCAATGGTGGTACACCATTGGTATGCGTACCAATGGTGATTTATACATTGGTTCTGTT
TTCTTGATGATTGTCGCTGCAGTCATGTTGTTTGCAGGTTGGCTTCATCTACAACCCAAA
TTTCGACCCAGCTTAGCCTGGTTTAGAGATGCTGAATCCCAAATGAACCACCACTTGGCA
GTTCTATTTGGTGCTAGCTCTTTGGGCTGGACAGGCCACTTAATCCACGTTGCTATTCCC
GAAGCTCGGGGTCAGCACGTAGGTTGGGATAACTTTCTGTCAACCATGCCTCACCCTGCT
GGTTTAGCGCCTTTCTTTACTGGGCGTTGGGGAGTTTATGCTCAAAACCCTGATACTGCT
GGTCATATTTTTGGAACTAGCGAAGGTGCTGGAACTGCGATTATTACCTTTATTGGCGGT
TTCCATCCCCAAACTGAAGCATTGTGGCTAACTGATATTGCCCACCACCATCTGGCTATT
GCTGTGATGTACATCATTGCTGGCCATATGTATCGAACTCAGTTCGGTATTGGGCATAGT
ATGAAAGAGATCCTAGAAGCACACACCCCTCCCAGCGGGATGTTGGGTGATGCGCACAAG
GGCCTTTATGACACTTACAATGAATCTCTACATTTCCAGTTAGGTTTCCACCTAGCTGCA
TTAGGTGTAATCACTTCTGTGGTTGCCCAACATATGTATTCATTGCCGTCATACGCTTTC
ATCTCTCAAGACCATGTCACACAAGCTGCGCTTTACACACATCACCAATATATTGCTGGA
ATTCTAGCAATTGGTGCTTTTGCGCATGGTGGTATCTTCTTTGTCCGAGATTACGATCCA
GAACGTAACAAGAACAACGTTCTTGCTCGTGCTCTTGAGCATAAAGAGGCGATTATCTCC
CACCTATCTTGGGTATCCATGTTCAGTGGTTTCCATACCCTTGGTGTTTATGTTCATAAC
GACACCGTGGTAGCTTTTGGTACTCCTGAGAAGCAAATTTTGGTTGAGCCAATCTTTGCG
CAATGGATTCAGGCAGCTCATGGCAAACTGCTCTTAGGATTTGAAACACTGCTTTCAAAT
CCTAATGGATTGGCTTATAACCCTCCTAACATTTCTCCTGATGTATTTGTTCCTGGATGG
GTTGAAGCAATGAACAACCCTGTTATCGGGCCGTTTATGTCTCAAGGGCCTGGTGACTTC
TTGGTTCATCATGGTATTGCCTTCAGTTTGCATGTCACCGTCTTAATCTGTGTCAAGGGT
TGTTTGGATGCCCGTGGTTCTAAACTGATGCCTGACAAGAAAGACTTTGGTTATAGCTTC
CCTTGTGATGGCCCCGGACGTGGCGGTACTTGTGATATCTCTGCTTGGGATTCCTTCTAC
CTTGCCTTCTTCTGGATGCTCAACACAATTGGTTGGATTGTCTTCTACTTCA ACTGGAAG
CATTTGGCTATCTGGTCTGGTAACGAAGCTCAGTTCAATACCAACTCTACTTATCTAATG
GGTTGGCTGCGAGACTACCTTTGGGGATACTCAGCTCAATTGATTAACGGTTACACACCA
TTTGGTGTAAATAGCCTGTCAGTTTGGGCTTGGATTTTCCTCTTAGGCCACCTCTGCTGG
GCGACTGGCTTCTTGTTCTTGATCTCCTGGAGAGGTTACTGGCAAGAGCTGATTGAGACT
CTCGTTTGGGCTCACCAGCGTACTCCCCTCGCCAACTTAGTGACATGGAAAGACAAGCCT
GTTGCTCTCTCTATCGTTCAAGGTCGCTTGGTGGGTTTAGTCCACTTTGCGGTTGGCTAT
TATGTGACCTACGCGGCTTTTGTGATTGGTGCAACAGCTCC TCTCGGCTAA
```

```
MATKFPSFSQDLAQDPTTRRIWYGIATVHDFETHDGMTEENLYQKIFATHFGHLSIIFLW
SAGHLFHVAWQGNFEQWIQDPLTIRPIAHAIWDPHLGDAATQAFTQAGASGPVDLCYSGL
YQWWYTIGMRTNGDLYIGSVFLMIVAAVMLFAGWLHLQPKFRPSLAWFRDAESQMNHHLA
VLFGASSLGWTGHLIHVAIPEARGQHVGWDNFLSTMPHPAGLAPFFTGRWGVYAQNPDTA
GHIFGTSEGAGTAIITFIGGFHPQTEALWLTDIAHHHLAIAVMYIIAGHMYRTQFGIGHS
MKEILEAHTPPSGMLGDAHKGLYDTYNESLHFQLGFHLAALGVITSVVAQHMYSLPSYAF
ISQDHVTQAALYTHHQYIAGILAIGAFAHGGIFFVRDYDPERNKNNVLARALEHKEAIIS
HLSWVSMFSGFHTLGVYVHNDTVVAFGTPEKQILVEPIFAQWIQAAHGKLLLGFETLLSN
PNGLAYNPPNISPDVFVPGWVEAMNNPVIGPFMSQGPGDFLVHHGIAFSLHVTVLICVKG
CLDARGSKLMPDKKDFGYSFPCDGPGRGGTCDISAWDSFYLAFFWMLNTIGWIVFYFN WK
HLAIWSGNEAQFNTNSTYLMGWLRDYLWGYSAQLINGYTPFGVNSLSVWAWIFLLGHLCW
ATGFLFLISWRGYWQELIETLVWAHQRTPLANLVTWKDKPVALSIVQGRLVGLVHFAVGY
YVTYAAFVIGATAPLG*
```

Supplementary Fig. 20-b: Assessment of the accuracy of DNA sequence of *psaB* in the database. The accuracy of the DNA sequence (accession number ABW27466.1) of the blue-colored region in the upper panel was confirmed. The corresponding amino acid sequence is shown in the lower panel. The codon and residue corresponding to the ligand of Acc in other organisms are shown in magenta. DNA sequencing was performed for forward- and reverse-reading twice, respectively, with the same sequence as that of accession number ABW27466.1.

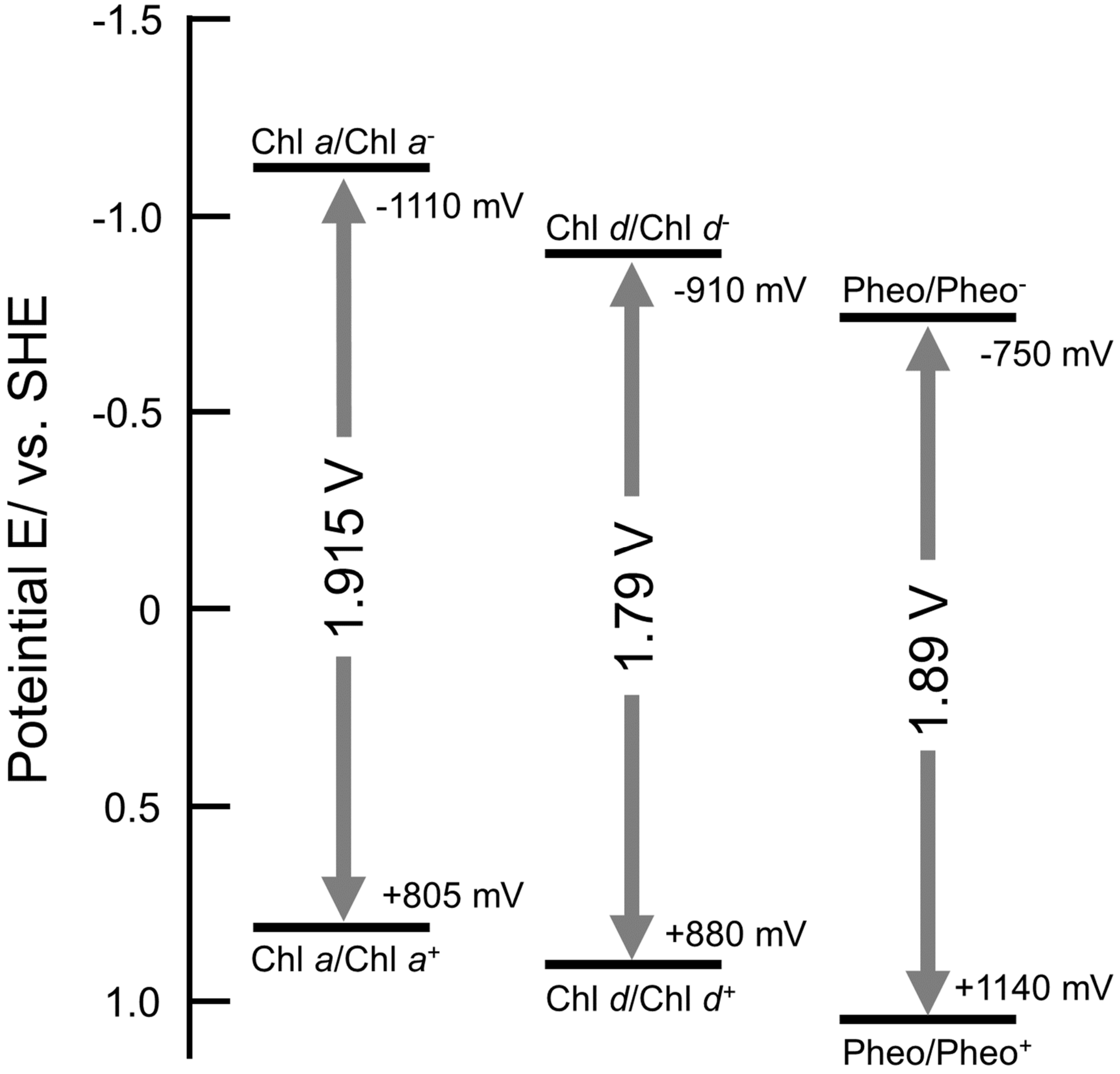

Supplementary Fig. 21: Schematic comparison of reduction potentials of Chl *a*, Chl *d*, and Pheo in acetonitrile.

The values refer to Kobayashi *et al*. (2013)[51] and Komatsu *et al*. (2014)[52].

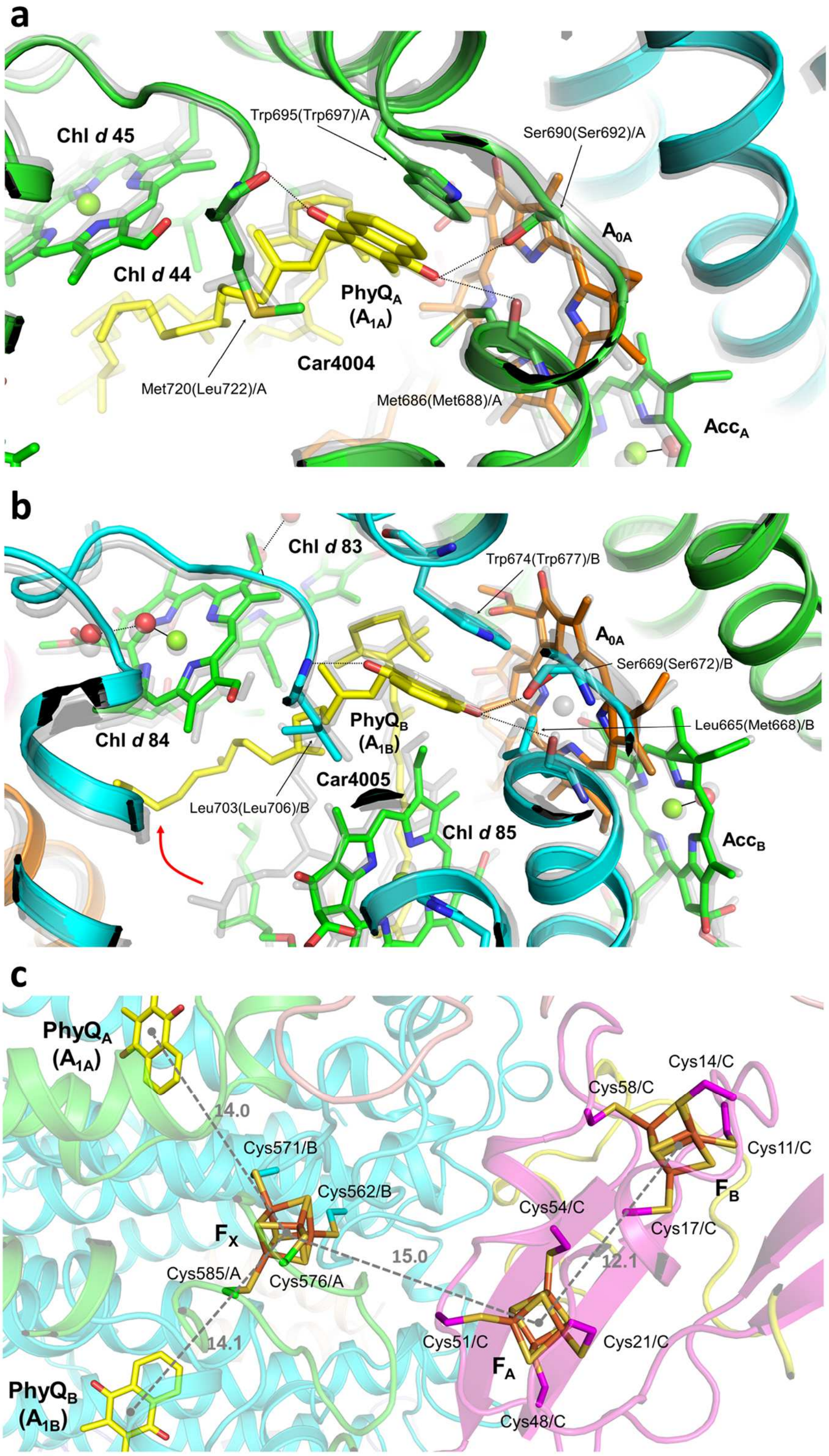

Supplementary Fig. 22: Interactions between cofactors and their surrounding amino acid residues in *A. marina* PSI.

(a) Interaction between $PhyQ_A$ and its surrounding structures. (b) Interaction between $PhyQ_B$ and its surrounding structures. (c) Distances (in Å) between cofactors and the interaction between the iron–sulfur clusters (F_X, F_A, and F_B) and their surrounding amino acid residues.

Supplementary Table 1: Subunit proteins in *A. marina* PSI.

Subunit	Chain ID	Length (residues)	Number of assigned amino acid residues	Model state	Main function in PSI
PsaA	aA, bA, cA	765	41 - 243, 272 - 753	Mainly assigned by each amino acid residue	Reaction center and electron transfer
PsaB	aB, bB, cB	748	3 - 291, 322 - 464, 511 - 736	Mainly assigned by each amino acid residue	Reaction center and electron transfer
PsaC	aC, bC, cC	82	2 - 81	Mainly assigned by each amino acid residue	Electron transfer
PsaD	aD, bD, cD	141	4 - 139	Mainly assigned by each amino acid residue	Binding site of ferredoxin
PsaE	aE, bE, cE	90	1 - 67	Mainly polyalanine model	Possibily ferredoxin docking
PsaF	aF, bF, cF	169	28 - 77, 84 - 165	Mainly polyalanine model	Binding site of plastocyanin or cytochrome *c*
PsaI	aI, bI, cI	34	3 - 32	Mainly assigned by each amino acid residue	Stabilization of oligomer
PsaJ	aJ, bJ, cJ	51	16 - 50	Assigned by each amino acid residue	Stabilization of PsaF
PsaK	aK, bK, cK	60	21 - 36, 61 - 77	Polyalanine model	State transition
PsaL	aL, bL, cL	155	5 - 151	Mainly assigned by each amino acid residue	Stabilization of oligomer
PsaM	aM, bM, cM	31	2 - 30	Assigned by each amino acid residue	Stabilization of oligomer

Supplementary Table 2: Subunit identification by mass spectrometry and Mascot.
Lane numbers correspond to those in Supplementary Fig. 6.

Assigned subunits	Protein Accession #	Protein Mass	Protein Score	Protein Matches
Lane 2				
PsaB	gi|158305849	82081	893	150
PsaA	gi|158305848	83333	951	168
PsaD	gi|158308489	15087	1031	281
PsaL	gi|158304848	15330	406	105
PsaF	gi|158304851	17815	339	217
PsaE	gi|158305894	9542	385	139
PsaC	gi|158305064	8810	167	100
PsaJ	gi|158304850	5893	16	1
Lane 3				
PsaB	gi|158305849	82081	720	133
PsaA	gi|158305848	83333	715	194
PsaD	gi|158308489	15087	746	220
PsaF	gi|158304851	17815	339	157
PsaL	gi|158304848	15330	344	119
PsaE	gi|158305894	9542	310	105
PsaC	gi|158305064	8810	210	98
PsaK	gi|158305041	9276	18	1
PsaJ	gi|158304850	5893	16	1

Supplementary Table 3: Statistics for data collection, processing, and refinement.

	#1 PSI trimer (EMDB-30420) (PDB 7COY)
**Data collection**	
Microscope	CRYO ARM 300
Imaging device	K2 summit
Voltage (kV)	300
Imaging mode	Counted
Software	JADAS
Grid condition	QF1.2/1.3Cu
	Au sputtered
Magnification	x60,000
Total exposure time (sec)	10
No. of frames	50
Electron exposure (e–/Å^2)	85.7
Defocus range (µm)	-0.6 - -1.8
Original pixel size (Å)	0.823
No. of total image sets	7,571
Duration (day)	9
**Data processing**	
No. of used image sets	4,237
Initial particles (no.)	774,416
Final particles (no.)	86,419
Pixel size for final map (Å)	1.08
Symmetry imposed	C3 (C1)
Map resolution (Å)	2.58 (2.97)
FSC threshold	0.143 (0.143)
B-factor for sharpening (Å^2)	-92.82 (-77.95)
Map resolution range (Å)	2.37 – 3.92 (2.63 – 4.00)
**Refinement**	
Initial model used (PDB code)	1JB0
Model resolution (Å)	2.58
FSC threshold	0.50
Model resolution range (Å)	2.58 - 204.0
Model composition	
Non-hydrogen atoms	579,66
Protein residues	44,574
Ligands	13,308
Waters	84
B factors (Å^2)	
Protein	100.9
Ligand	94.5
Water	29.1
R.m.s. deviations	
Bond lengths (Å)	0.016
Bond angles (°)	1.734
Validation	
MolProbity score	1.96
Clashscore	11.4
Poor rotamers (%)	0.90
Ramachandran plot	
Favored (%)	94.4
Allowed (%)	5.6
Disallowed (%)	0.0

Supplementary Table 4: Evaluation of the agreement between EM map and final model.

	CC*	Q-score#	Estimated resolution (Å) by Q-score#
Overall structure (PSI trimer)	0.80	0.65	2.6
Protein subunit (Chain ID)			
PsaA (aA, bA, cA)	0.79	0.69	2.4
PsaB (aB, bB, cB)	0.82	0.69	2.4
PsaC (aC, bC, cC)	0.77	0.65	2.6
PsaD (aD, bD, cD)	0.74	0.61	2.9
PsaE (aE, bE, cE)	0.54	0.56	3.2
PsaF (aF, bF, cF)	0.68	0.51	3.5
Psa27 (aI, bI, cI)	0.82	0.75	2.1
PsaJ (aJ, bJ, cJ)	0.71	0.56	3.2
PsaK (aK, bK, cK)	0.53	0.44	3.9
PsaL (aL, bL, cL)	0.82	0.75	2.1
PsaM (aM, bM, cM)	0.70	0.62	2.8
Cofactor			
Ligands	0.77	0.72	1.98
Waters	0.83	0.87	1.42

*CC: Correlation coefficient between the refined structure model of PSI and the cryo-EM map.

#Q-score: An index showing resolvability of atoms, amino acid residues, and ligands assigned in a cryo-EM map (Local resolution map). Resolution (Å) is estimated by the formula (Q-score = −0.1775 × Resolution (Å) + 1.1192) according to Pintilie *et al*. (2020)[73].

Supplementary Table 5: Sequence identity between the subunits of PSI from *A. marina* and those from other species.

The values of identity were calculated using MODELLER for comparative protein structure modeling.

	T. elongatus	*Synechocystis*	*H. hongdechloris*
Oligomerization	Trimer	Trimer	Trimer
PDB code	1JB0	5OY0	6KMW, 6KMX
PsaA	75.6	75.1	72.6
psaB	77.2	78.2	77.2
PsaC	98.8	96.2	96.3
PsaD	76.1	71.9	70.5
PsaE	72.0	65.2	77.9
PsaF	62.8	50.3	N/A
Psa27 (vs PsaI)	29.4	29.4	23.5
PsaJ	53.7	55.0	N/A
PsaK1 (PsaK2)	35.0 (44.6)	20.0 (37.5)	33.3 (41.9)
PsaL	40.5	38.6	40.5
PsaM	54.8	64.5	51.6

	Anabaena	*C. reinhardtii*	*C. merolae*
Oligomerization	Tetramer	Monomer	Monomer
PDB code	6JEO	6JO6	5ZGH
PsaA	75.0	74.8	72.6
psaB	76.8	74.2	72.4
PsaC	97.5	87.7	92.6
PsaD	40.3	54.0	7.2
PsaE	82.9	53.4	42.7
PsaF	54.9	39.4	38.9
Psa27 (vs PsaI)	38.2	29.4	37.5
PsaJ	55.1	58.5	57.9
PsaK1 (PsaK2)	38.3 (19.8)	3.3 (25.6)	8.3 (40.0)
PsaL	45.8	38.6	42.9
PsaM	54.8	N/A	34.5

*N/A: Not assigned.

Supplementary Table 6: Pigment composition of PSI trimer relative to Chl *d*' determined by HPLC analysis.

Amounts of each pigment were determined by HPLC analysis and their molar ratios to Chl *d*' are presented. Standard errors are shown in parentheses.

	Zeaxanthin	Chl *d*	Chl *a*	Phe *a*	α-carotene	PhyQ
Lot #1 (n=3)	1.18 (±0.022)	67.7 (±0.45)	1.17 (±0.005)	1.94 (±0.016)	10.0 (±0.018)	2.06 (±0.172)
Lot #2 (n=3)	0.939 (±0.015)	65.8 (±0.31)	1.12 (±0.008)	1.94 (±0.016)	10.2 (±0.093)	2.05 (±0.076)
Lot #3 (n=4)	1.03 (±0.037)	66.2 (±1.80)	1.13 (±0.034)	1.93 (±0.052)	9.01 (±0.55)	1.83 (±0.142)
Lot #4 (n=3)	0.95 (±0.016)	66.2 (±1.39)	1.14 (±0.018)	1.96 (±0.045)	9.97 (±0.42)	1.81 (±0.103)
Lot #5 (n=2)	1.32 (±0.066)	69.3 (±5.21)	0.924 (±0.058)	1.83 (±0.152)	11.0 (±0.847)	2.00 (±0.219)
mean values	1.09 (±0.074)	67.0 (±0.66)	1.10 (±0.044)	1.92 (±0.022)	10.0 (±0.32)	1.94 (±0.053)

Source data are provided as a Source Data file.

Supplementary Table 7: Primers used for DNA sequences.

Primer	Sequence
psaA-F	5´-GTACAACTGCATCTCAATTG-3´
psaA-R	5´-CTATCCTAATGCGAGAATTC-3´
psaB-F	5´-CCTTGCCTTCTTCTGGATGC-3´
psaB-R	5´-TTAGCCGAGAGGAGCTGTTG-3´
