# Supplementary material for: Structure of the far-red light utilizing photosystem I of Acaryochloris marina
Source: Nat Commun. 2021 Apr 20;12:2333. doi: 10.1038/s41467-021-22502-8 (PMC8058080; doi:10.1038/s41467-021-22502-8)
Supplement: Supplementary file 2 — Description of Additional Supplementary Files [file 41467_2021_22502_MOESM2_ESM.pdf]

Description of additional supplementary information

Title: Supplementary Data 1

Description: Numbering of the identified pigments in PSI.
