# Supplementary material for: Structure of the far-red light utilizing photosystem I of Acaryochloris marina
Source: Nat Commun. 2021 Apr 20;12:2333. doi: 10.1038/s41467-021-22502-8 (PMC8058080; doi:10.1038/s41467-021-22502-8)
Supplement: Supplementary file 5 — Reporting Summary [file 41467_2021_22502_MOESM5_ESM.pdf]

nature research

Corresponding author(s): Yasuhiro Kashino, Koji Yonekura, Keisuke Kawakami

Last updated by author(s): Mar 6, 2021

Reporting Summary

Nature Research wishes to improve the reproducibility of the work that we publish. This form provides structure for consistency and transparency in reporting. For further information on Nature Research policies, see our Editorial Policies and the Editorial Policy Checklist.

Statistics

For all statistical analyses, confirm that the following items are present in the figure legend, table legend, main text, or Methods section.

n/a	Confirmed	
☐	☒	The exact sample size (*n*) for each experimental group/condition, given as a discrete number and unit of measurement
☒	☐	A statement on whether measurements were taken from distinct samples or whether the same sample was measured repeatedly
☒	☐	The statistical test(s) used AND whether they are one- or two-sided<br>*Only common tests should be described solely by name; describe more complex techniques in the Methods section.*
☒	☐	A description of all covariates tested
☒	☐	A description of any assumptions or corrections, such as tests of normality and adjustment for multiple comparisons
☒	☐	A full description of the statistical parameters including central tendency (e.g. means) or other basic estimates (e.g. regression coefficient) AND variation (e.g. standard deviation) or associated estimates of uncertainty (e.g. confidence intervals)
☒	☐	For null hypothesis testing, the test statistic (e.g. *F*, *t*, *r*) with confidence intervals, effect sizes, degrees of freedom and *P* value noted<br>*Give P values as exact values whenever suitable.*
☒	☐	For Bayesian analysis, information on the choice of priors and Markov chain Monte Carlo settings
☒	☐	For hierarchical and complex designs, identification of the appropriate level for tests and full reporting of outcomes
☒	☐	Estimates of effect sizes (e.g. Cohen's *d*, Pearson's *r*), indicating how they were calculated

Our web collection on statistics for biologists contains articles on many of the points above.

Software and code

Policy information about availability of computer code

Data collection	JEOL Automatic Data Acquisition System (JADAS), Shimadzu LabSolutions LC/GC (Release 5.82)
Data analysis	MotionCor2 (version 1.1.3), CTFFIND4 (version 4.1.10), RELION-3.1beta, cryoSPARC (version 2.12.0), MODELLER (version 9.23), UCSF Chimera (version 1.13), CryoFit1, Phenix (version 1.19-4092), COOT (version 0.9.2), refmac5 (in CCP-EM 1.5.0), ModelZ, MapQ (version 1.6.4), PyMOL (version 2.4.0), Electronic Ligand Bond Builder and Optimization Workbench (eLBOW), MagicPlot (ver. 2.9.3)

For manuscripts utilizing custom algorithms or software that are central to the research but not yet described in published literature, software must be made available to editors and reviewers. We strongly encourage code deposition in a community repository (e.g. GitHub). See the Nature Research guidelines for submitting code & software for further information.

Data

Policy information about availability of data

All manuscripts must include a data availability statement. This statement should provide the following information, where applicable:
- Accession codes, unique identifiers, or web links for publicly available datasets
- A list of figures that have associated raw data
- A description of any restrictions on data availability

DATA AVAILABILITY
Atomic coordinates and cryo-EM maps for the reported structure of Acaryochloris marina PSI have been deposited in the Protein Data Bank under accession codes 7COY [https://www.rcsb.org/structure/7COY], and in the Electron Microscopy Data Bank under accession codes EMD-30420, respectively. Other data are available from the corresponding authors upon reasonable request.
Source data are provided with this paper for Supplementary Figs. 4b, 5, 6, 8, and Supplementary Table 6.

Field-specific reporting

Please select the one below that is the best fit for your research. If you are not sure, read the appropriate sections before making your selection.

☒ Life sciences ☐ Behavioural & social sciences ☐ Ecological, evolutionary & environmental sciences

For a reference copy of the document with all sections, see nature.com/documents/nr-reporting-summary-flat.pdf

Life sciences study design

All studies must disclose on these points even when the disclosure is negative.

Sample size	DNA sequencing shown in Supplementary Fig. 19-a and b was performed for forward- and reverse-reading twice, respectively. This means that the accuracy of codons corresponding to the ligand of Accs in A. marina PSI were confirmed four times by comparing with the sequences obtained from database (accession numbers ABW27465.1 and ABW27466.1). The sample size is reasonable since exactly the same sequences were obtained five times which is larger than three times.<br>Pigment composition by HPLC analysis (Supplementary Table 7 and Supplementary Fig. 15) was performed using five independent samples. The sample size is reasonable since it is larger than three times with low standard errors (±1.0% at most).<br>Sucrose density gradient centrifugation and following BN-PAGE (Supplementary Fig. 4) and SDS-PAGE (Supplementary Fig. 6) were performed every time upon purification of PSI, which is listed in Supplementary Table 7. The sample size is reasonable as the pigment composition was obtained with low standard errors (±1.0% at most) from these purified PSI.<br>BN-PAGE (Supplementary Fig. 5) were performed at least twice to make display figures. Furthermore, after additional three months incubation of those samples at 0oC under the dark gave essentially the same stability. Therefore, the sample size seems to be reasonable.<br>Based on Thon-ring patterns, 4,237 images of cryo-EM from data 1 and 2 were selected for particle picking. After 2D and 3D classifications, 86,419 particles were finally selected from the images. The sample size is reasonable since it yielded a 2.59-Å resolution map with C3 symmetrization.
Data exclusions	90 particles out of 86,509 particles were excluded before final 3D refinement since they showed unrealistic defocus values through Bayesian polishing for correction of particle-based beam-induced motion and contrast transfer function refinement of particle-based defoci and optics-group-based high-order astigmatisms.
Replication	At least five independently purified samples gave essentially the same result for sucrose density gradient centrifugation, BN-PAGE profile, SDS-PAGE profile, pigment composition.<br>Two independently purified samples gave fundamentally the same structure.
Randomization	The most initial 2D classification was performed free of reference meaning random classification with RELION-3.1beta using manually selected good PSI particles excluding apparently large/small or irregular particles. The following classification and refinement processes were basically along with standard routine for structure determination.
Blinding	Blinding for purification of PSI and the following biological experiment is not relevant since PSI was the only target among several protein complexes in this work.<br>The most initial 2D classification was performed free of reference meaning blinding classification with RELION-3.1beta.

Reporting for specific materials, systems and methods

We require information from authors about some types of materials, experimental systems and methods used in many studies. Here, indicate whether each material, system or method listed is relevant to your study. If you are not sure if a list item applies to your research, read the appropriate section before selecting a response.

Materials & experimental systems

n/a	Involved in the study
☒	☐ Antibodies
☒	☐ Eukaryotic cell lines
☒	☐ Palaeontology and archaeology
☒	☐ Animals and other organisms
☒	☐ Human research participants
☒	☐ Clinical data
☒	☐ Dual use research of concern

Methods

n/a	Involved in the study
☒	☐ ChIP-seq
☒	☐ Flow cytometry
☒	☐ MRI-based neuroimaging
